# Identification and validation of reference genes for qRT-PCR analysis in mulberry (*Morus alba* L.)

**DOI:** 10.1371/journal.pone.0194129

**Published:** 2018-03-15

**Authors:** Fanwei Dai, Xiting Zhao, Cuiming Tang, Zhenjiang Wang, Zheshi Kuang, Zhiyi Li, Jing Huang, Guoqing Luo

**Affiliations:** 1 Sericultural & Agri-Food Research Institute, Guangdong Academy of Agricultural Sciences, Guangzhou, China; 2 Key Laboratory of Urban Agriculture in South China, Ministry of Agriculture, Guangzhou, China; 3 College of Life Sciences, Henan Normal University, Xinxiang, China; Nazarbayev University, KAZAKHSTAN

## Abstract

Mulberry (*Morus alba* L.) is an important economic tree species in many countries. Quantitative real time PCR (qRT-PCR) has become a widely used method for gene expression studies in plants. A suitable reference gene is essential to ensure accurate and reliable results for qRT-PCR analyses. However, no reports describing the selection of reference genes have been published for mulberry. In this work, we evaluated the stability of twenty candidate reference genes in different plant tissues and under different stress conditions by qRT-PCR in mulberry using algorithms in two programs—geNorm and NormFinder. The results revealed that *TUB2*, *UBI4*, *ACTIN3* and *RPL4* were ranked as the most stable reference genes in the samples subsets, whereas *EF1α4* and *TUB3*showed the least stability with both algorithms. To further validate the stability of the reference genes, the expression patterns of six genes of mulberry were analyzed by normalization with the selected reference genes. Our study will benefit future analyses of gene expression in mulberry.

## Introduction

Mulberry (*Morus alba* L.) is widely cultivated as an economic tree species in most developing countries in Asia, as it is the sole food source of the domesticated silkworm (*Bombyx mori* L.). In addition, mulberry has been used as a food product and in herbal medicines for a long time, as it can be used as a pharmaceutical treatment for hyperglycemia, inflammation, hypertension, and fever [1,2]. The mulberry genome has recently been sequenced, and high-throughput sequencing has been used in several mulberry studies [3–5]. Genome sequence and large-scale transcriptome data have greatly facilitated molecular studies in mulberry.

Gene expression analysis is widely used in many fields of plant biological research including in development and in responses to stress. Quantitative real-time polymerase chain reaction (qRT–PCR) has become the most popular approach for gene expression studies because of its rapidity, sensitivity, and specificity [6]. However, the reliability of the results obtained from qRT-PCR depends on accurate normalization using stably expressed reference genes [7]. Thus, it is necessary to select appropriate reference genes with minimal variability during the experimental conditions before qRT-PCR analysis.

In plants, some reference genes have been reported, but the stability of reference genes in different plant species is inconsistent [8–10]. *TUB-B*, *TUB-A*, and *UBC* are the most stable reference genes in celery [9]. *NTB* and *TIP41* are most stable in bamboo [11]. In addition, the expression of reference genes varies under different experimental treatments. In a study of switchgrass, *eEF-1α* and *ACT12* were most stable across all tissue samples, whereas *CYP5* and *FTHS4* were the most suitable reference genes during seed development under abiotic stress conditions as determined by NormFinder [10]. To date, a systematic study validating reference genes has not been reported in mulberry. Hence, it is necessary to identify the suitable reference genes in various tissues and under different abiotic stress conditions, which will be helpful for the accurate and reliable analysis of gene expression in this plant.

In this study, we examined the stability of twenty candidate reference genes (three *ACTIN* genes, three *TUB* genes, three *UBI* genes, three *EF1α* genes, two *GAPDH* genes, two *MDH* genes, two *RPL* genes, one *CYP* gene, and one *PP2A* gene) for use in qRT-PCR expression studies in mulberry. The expression patterns of these reference genes were quantified in various tissues and under different abiotic stress conditions using both geNorm and NormFinder algorithms [12,13]. In addition, three chalcone synthase genes of mulberry (*MaCHS5*, *MaCHS6*, *MaCHS7*) and three plant hormone related genes (*MaERF*, *MaDELLA*, *MaJAZ*) were used to evaluate the reliability of the most stable reference genes identified in this work.

## Results

### Amplification specificity and efficiency for each candidate reference gene

To identify suitable reference genes for mulberry, 20 candidate reference genes were selected based on previous reports of control genes used in various plant species [10,14,15]. The gene sequences of *ACTIN2* and *ACTIN3* were obtained from the National Center for Biotechnology Information (NCBI, USA). The sequences of the eighteen other candidate reference genes were selected from our transcriptome database that was generated via high-throughput Illumina sequencing (S1 Table) [4], and the expression of these eighteen candidate reference genes was relatively stable at all treatment times of three mulberry varieties after infection with *Ralstonia solanacearum* in our previous study (S2 Table) [16]. The products of these twenty genes are associated with a wide variety of biological functions.

**Fig 1:**
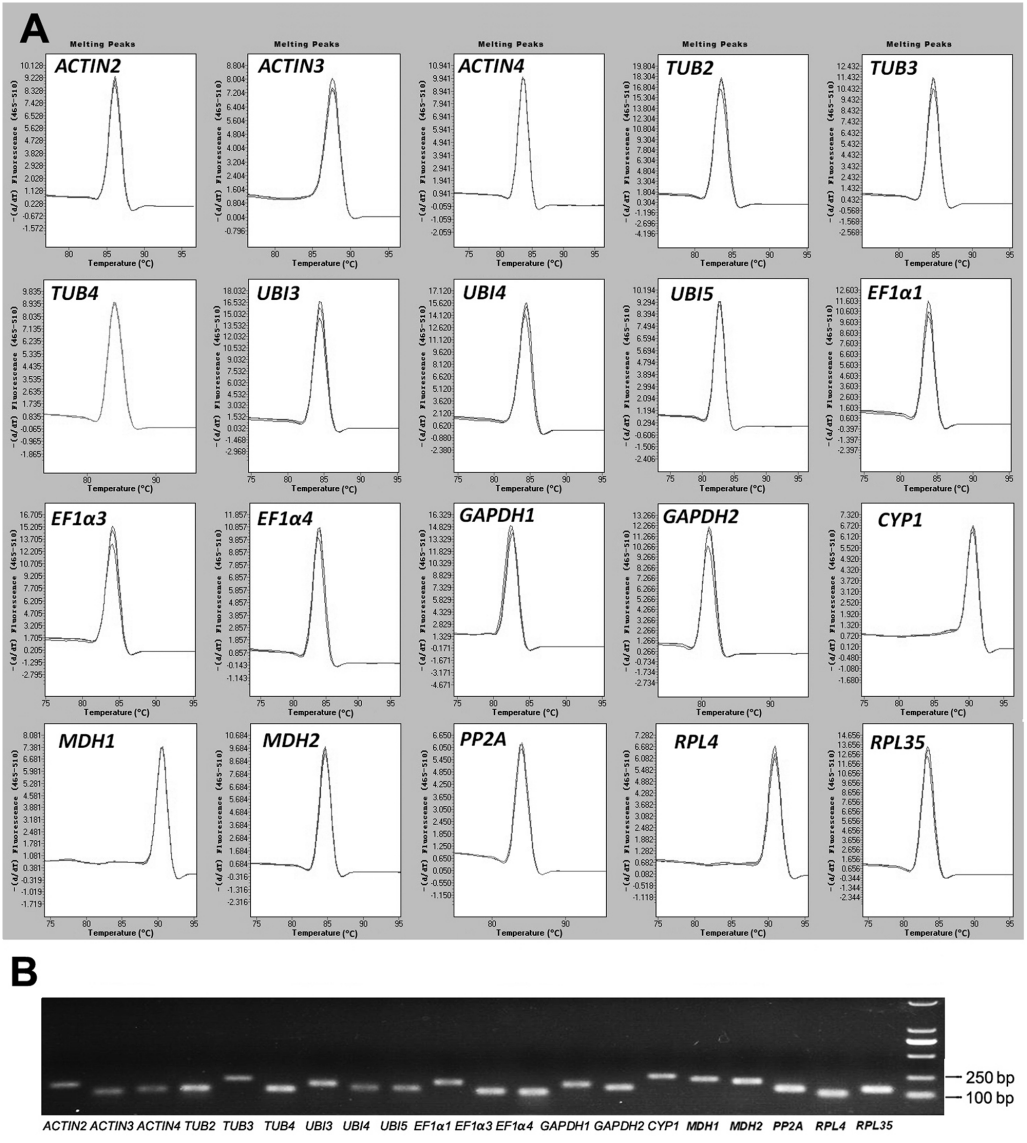
Specificity of qRT-PCR amplification for the 20 candidate reference genes. (A) Melting curves for each gene show a single peak. (B) Agarose gel showing amplification of a specific PCR product of the expected size for each gene tested in this study.

To check the specificity of the primers for these candidate reference genes, we performed qRT-PCR melting curve and agarose gel electrophoresis analyses. The product of each primer pair was a single peak in the melting curve and a single band with the expected size after agarose gel electrophoresis and polyacrylamide gelelectrophoresis (PAGE) electrophoresis (Fig 1 and S1 Fig). The qRT-PCR amplification efficiency for the fifteen candidate reference genes varied from 95.3% (*ACTIN3*) to 105.7% (*EF1α1*), and correlation coefficient (R^2^) values ranged from 0.990 to 0.999 (Table 1).

**Table 1:** Information about the candidate reference genes.

Gene name	Gene description	Accession number/RNA-Seq number	Primer sequence (5′–3′)	Amplicon length (bp)	Amplificationefficiency (%)	R^2^
*ACTIN2*	Morus alba actin 1 (ACT1)	HQ163776	F:GGCCATTCAAGCCGTTCTTTCTCTA R:AATTTCATCAAGTGGTCGGTGAGAT	174	105.6	0.992
*ACTIN3*	Morus alba actin 3 (ACT3)	HQ163775	F: GAGGGCCGTGTTCCCCAGCATCGTC R: TCTTTTTGATTGAGCCTCATCCCCT	106	95.3	0.996
*ACTIN4*	Actin	Unigene23275/ KT793030	F: TGTTGCTCCACCAGAGAGAAAGTAC R: GGACAATTGATGGACCAGACTCG	125	95.6	0.992
*TUB2*	Tubulin beta-3 chain	CL1595.Contig6	F: GGATACCCAATAATGTGAAGTCTAGC R: CGTGAACTGCTCGCTGACCCTC	128	99.3	0.996
*TUB3*	Tubulin beta-1 chain protein	CL8311.Contig1	F: CAAGGGTCACTACACTGAGGGAGCA R: TCGGTGATGGGAACACAGAGAATGT	215	100.4	0.994
*TUB4*	Tubulin alpha chain	CL2672.Contig2	F: CCAAACAGACCAAGAAGAGGTAGAA R: CATGCTCAAGGCAGTAAAGCTCC	120	97.1	0.998
*UBI3*	Ubiquitin-like protein	Unigene18627	F: ACAGGCACGAGAGCCGACAAGATT R: CCAAGCTCCACTAGCGTATAGAACA	167	101.0	0.998
*UBI4*	Ubiquitin-conjugating enzyme E2	Unigene5350	F: TCTCTAACCCCGAGAAATCTCTCAC R: ACGACACTCGATCCGCCTGAGC	129	101.3	0.999
*UBI5*	ubiquitin-activating enzyme E1	CL1942.Contig2	F: ACGGTGCATTCCTTTCCACAC R: TTCTCATTGACGTCGCATACTCAC	144	99.2	0.997
*EF1α1*	Elongation factor 1-alpha	CL4198.Contig2	F: TAAATATAGGACAAAGCCATTTCCC R: CGACTTTCCAGAGTCAACGTGG	194	105.7	0.993
*EF1α3*	Elongation factor 1-alpha	CL272.Contig1	F: TTGGTGTCATAAAGAGCGTTGAGAA R: ATGAAGAAGAAAATCTCGTGGCAAA	115	102.4	0.997
*EF1α4*	Elongation factor 1-alpha	CL155.Contig3	F: ACGGTGGCGACAGGGCGTGT R: ACATCTCAACCCCAGTAACTGTGGT	109	97.5	0.997
*GAPDH1*	Glyceraldehyde-3-phosphate dehydrogenase	CL3772.Contig5	F: ATCCCTAACATTGGCATTTCCTCG R: CCTACCGATTCTTCCAAAACCGTTG	169	99.1	0.993
*GAPDH2*	Glyceraldehyde-3-phosphate dehydrogenase	CL131.Contig2	F: TCCAGGGTTTGAAGGACAGTGGC R: CTTCTCATTCCAAAGCGCATCATCC	138	95.7	0.996
*CYP1*	Cyclophilin	Unigene15318	F: CTTTAGCCATTTCTCATTTTCAGTG R: GGTGGAAGGACGATCCCTTGTAG	240	98.2	0.999
*MDH1*	Malate dehydrogenase	Unigene16243	F: GCGTCGTGGCTACTCCGTTCACT R: TCTCCTCACCCGCATATCCTTTA	215	98.1	0.999
*MDH2*	Malate dehydrogenase	Unigene15890	F: GGAGGATGCCTTGACGGGTATGG R: GCCGCAATGGGAACAGTGGAG	198	99.7	0.990
*PP2A2*	Protein phosphatase 2A	Unigene31643	F: TGAATCGAAGAATGAGATGTGTTCC R: AAAAATAGCAGTTTGTTTAATATGCC	138	96.6	0.997
*RPL4*	Ribosomal protein L4-like	Unigene26991	F: GTCTCCAATGGCAGCCACAGC R: CATCGGGTAGGGCGACGGTTT	104	99.1	0.991
*RPL35*	Ribosomal protein L35	CL8011.Contig2	F: TCTCCGTCCCAAGAAGACCAG R: TGAAATGGAACCCACCCTACA	143	96.0	0.995

### Expression profiles of the candidate reference genes

**Fig 2:**
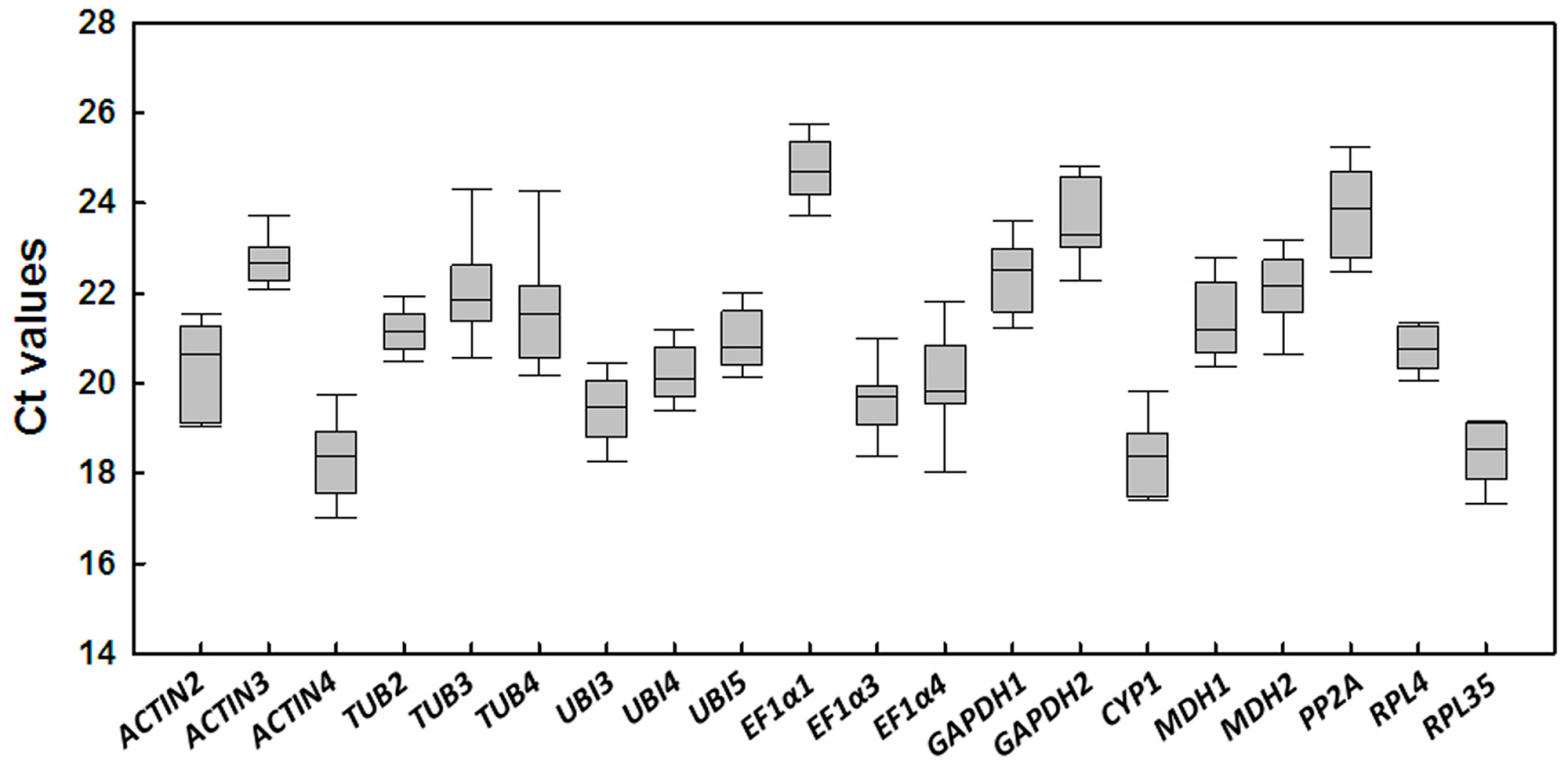
Cycle threshold (Ct) values of the candidate reference genes across the experimental samples. Box-plot graph of Ct value shows the median value as a line across the box. Lower and upper boxes indicate the 25th percentile to the 75th percentile, and whiskers indicate the ranges for all samples.

Expression levels of the 20 candidate reference genes were measured in the 10 different samples with three biological repeats obtained from leaves, stems, roots, green fruit, and red fruit of each plant under normal growth conditions and from leaves from plants under different abiotic stress conditions. The cycle threshold (Ct) values in the qRT-PCR reactions were used to identify the differences in transcript expression levels, with lower Ct values indicating higher transcript abundance and vice versa. We investigated the Ct values of all candidate reference genes within the set of samples, and variation in the expression of these genes is shown in Fig 2. A wide range of expression levels of these genes in all tested samples was observed. Most of the candidate reference genes had Ct values that range from 18 to 24. *EF1α1* had the highest mean Ct value at 24.7 and thus had the lowest level of expression among the candidate reference genes. In contrast, *ACTIN4*, with the lowest average Ct value of 18.3, had the highest level of transcript abundance. The variability of Ct values in all samples was lowest for *RPL4* (1.40) and *TUB2* (1.80), whereas *TUB3* (5.76) and *TUB4* (5.16) showed the largest variation in gene expression (Fig 2). None of the candidate reference genes showed a constant expression level among all samples. Therefore, it was necessary to carry out further analysis to select the most suitable reference genes for normalizing gene expression under particular experimental conditions.

### Expression stability analysis

Two commonly used analysis programs, geNorm and NormFinder, were applied to evaluate the expression stability of the candidate reference genes [12,13]. We used this software to analyze gene expression stability across different subsets of samples: 1) all samples, 2) tissue samples, 3) abiotic stress samples, and 4) green fruit and red fruit samples.

**Fig 3:**
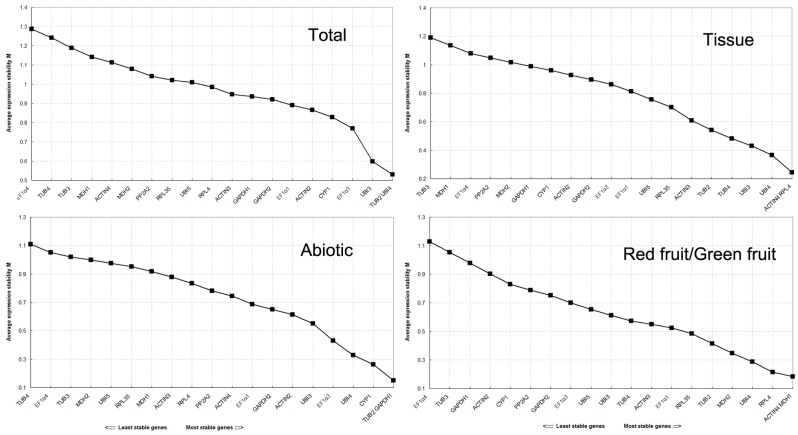
Gene expression stability values (M) of the candidate reference genes calculated by geNorm. Ranking of gene expression stability was performed in all the samples, in abiotic stress samples, in tissue samples, and in green fruit and red fruit samples. The lowest M value indicates the most stable gene, whereas the highest value represents the most highly variable gene.

**Fig 4:**
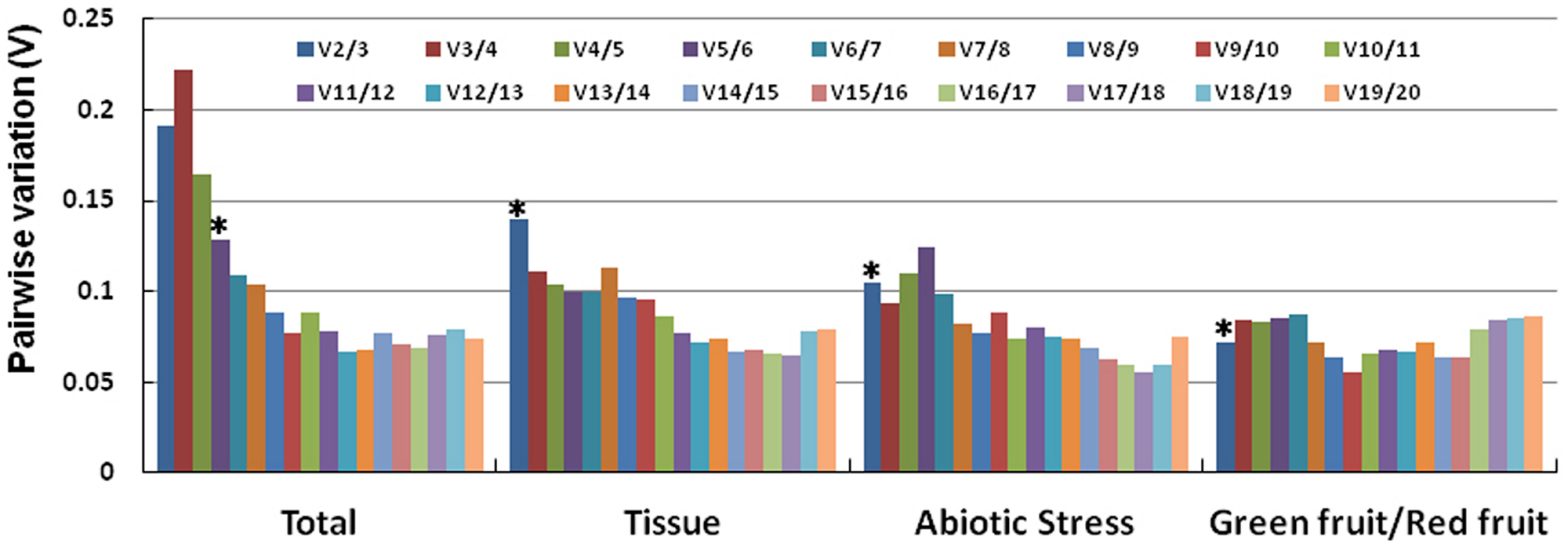
Pairwise variation (V) analysis of the candidate reference genes. The pairwise variation Vn/(n + 1) was analyzed between the normalization factors NFn and NFn+1 using geNorm software. Vn/(n + 1) < 0.15 indicates that the inclusion of an additional reference gene is not required. Asterisks indicate the optimal number of reference genes required for normalization.

The ranks of the candidate reference genes are presented in Fig 3 according to their average expression stability value (M) using geNorm. When all the samples were taken together, *TUB2* and *UBI4* showed the lowest average expression stability value (M = 0.531), and *EF1α4* showed the highest value (M = 1.286), indicating that *TUB2* and *UBI4* had the most stable expression and *EF1α4* had the highest level of expression variation among the 20 candidate genes. Pairwise variation was also calculated using geNorm (Fig 4). Pairwise variation analysis showed the optimal number of reference genes required for normalization, with a cut-off value of 0.15 being widely accepted as the criterion for determining a suitable number of reference genes [12]. For all samples, the value of V4/5 was 0.165 and of V5/6 was 0.128. The value of V5/6 was lower than the cut-off value of 0.15, indicating that the use of the five most stable reference genes was required for accurate normalization. Meanwhile, stability of expression for candidate reference genes was evaluated by NormFinder software according to the intra- and intergroup variations for normalization factor calculations. As shown in Table 2, *TUB2* and *UBI5* with the lowest stability value of 0.019 were identified as the best combination by NormFinder, and the most stable gene was *TUB2* (V = 0.027), whereas *EF1α4*, with the highest stability value of 0.068, was identified as the least stable reference gene.

**Table 2:** Expression stability values for candidate reference genes as calculated by the NormFinder software.

Total		Tissue		Abiotic		Green Fruit/Red Fruit	
**Ranking**	**Stability value**	**Ranking**	**Stability value**	**Ranking**	**Stability value**	**Ranking**	**Stability value**
*TUB2*	0.027	*ACTIN3*	0.016	*TUB2*	0.027	*EF1α1*	0.008
*ACTIN3*	0.030	*TUB2*	0.024	*GAPDH1*	0.030	*RPL35*	0.015
*UBI4*	0.035	*EF1α1*	0.030	*PP2A2*	0.033	*TUB2*	0.015
*RPL4*	0.036	*RPL4*	0.036	*UBI4*	0.033	*ACTIN3*	0.017
*EF1α1*	0.036	*UBI4*	0.036	*ACTIN4*	0.035	*PP2A2*	0.021
*GAPDH1*	0.041	*UBI5*	0.036	*RPL4*	0.035	*MDH1*	0.024
*UBI5*	0.041	*GAPDH2*	0.037	*GAPDH2*	0.036	*UBI4*	0.026
*GAPDH2*	0.042	*UBI3*	0.039	*CYP1*	0.039	*MDH2*	0.027
*ACTIN2*	0.045	*EF1α3*	0.040	*EF1α1*	0.039	*ACTIN4*	0.030
*PP2A2*	0.045	*RPL35*	0.040	*MDH1*	0.041	*UBI5*	0.031
*UBI3*	0.046	*ACTIN2*	0.043	*UBI3*	0.042	*RPL4*	0.033
*RPL35*	0.048	*GAPDH1*	0.046	*ACTIN3*	0.043	*EF1α3*	0.035
*CYP1*	0.050	*MDH2*	0.048	*ACTIN2*	0.043	*TUB4*	0.035
*MDH2*	0.051	*ACTIN4*	0.048	*UBI5*	0.045	*GAPDH2*	0.040
*EF1α3*	0.054	*PP2A2*	0.050	*TUB3*	0.045	*ACTIN2*	0.047
*MDH1*	0.054	*TUB4*	0.050	*RPL35*	0.049	*UBI3*	0.050
*ACTIN4*	0.058	*CYP1*	0.057	*MDH2*	0.049	*CYP1*	0.057
*TUB3*	0.059	*TUB3*	0.061	*EF1α3*	0.050	*GAPDH1*	0.068
*TUB4*	0.060	*MDH1*	0.062	*TUB4*	0.055	*TUB3*	0.072
*EF1α4*	0.068	*EF1α4*	0.063	*EF1α4*	0.067	*EF1α4*	0.081
**Best combination**	**Stability value**	**Best combination**	**Stability value**	**Best combination**	**Stability value**	**Best combination**	**Stability value**
*TUB2/ UBI5*	0.019	*ACTIN3/ TUB2*	0.011	*UBI4/ RPL4*	0.013	*TUB2/ EF1α1*	0.005
**Most stable**	**Stability value**	**Most stable**	**Stability value**	**Most stable**	**Stability value**	**Most stable**	**Stability value**
*TUB2*	0.027	*ACTIN3*	0.016	*TUB2*	0.027	*EF1α1*	0.008

In the tissue samples subset, which included root, leaf, stem, green fruit, and red fruit of mulberry under normal growth conditions, *ACTIN4* and *RPL4* were identified as the best pair with an M value of 0.243 by geNorm, whereas *TUB3* was the worst gene (Fig 3). Only two reference genes were sufficient for normalization because the pairwise variation value V2/3 was lower than 0.15 for this subset (Fig 4). NormFinder identified *ACTIN3* and *TUB2* as the best pair for the tissue samples subset, whereas *ACTIN3* and *EF1α4* were identified as the best and the worst reference genes, respectively (Table 2).

For abiotic stress samples, *TUB2* and *GAPDH1* were the best candidates (M = 0.151) for normalization by geNorm, whereas *TUB4* was the worst gene (M = 0.881) (Fig 3). Similarly, two control genes were satisfactory for normalization with a V2/3 value of 0.104 (Fig 4). *UBI4* and *RPL4* were identified as the most suitable pair by NormFinder, whereas *TUB2* and *EF1α4* were identified as the best and the worst reference genes, respectively (Table 2).

In the green fruit and red fruit sample subset, *ACTIN4* and *MDH1* were identified as the most stable genes by geNorm (Fig 3), whereas *TUB2* and *EF1α1* were identified as the most stable genes by NormFinder (Table 2). Two reference genes were sufficient for normalization because the pairwise variation value V2/3 was 0.072 for this subset by geNorm (Fig 4). Both algorithms identified *EF1α4* as the least stable gene.

### Reference gene validation in gene expression study

**Fig 5:**
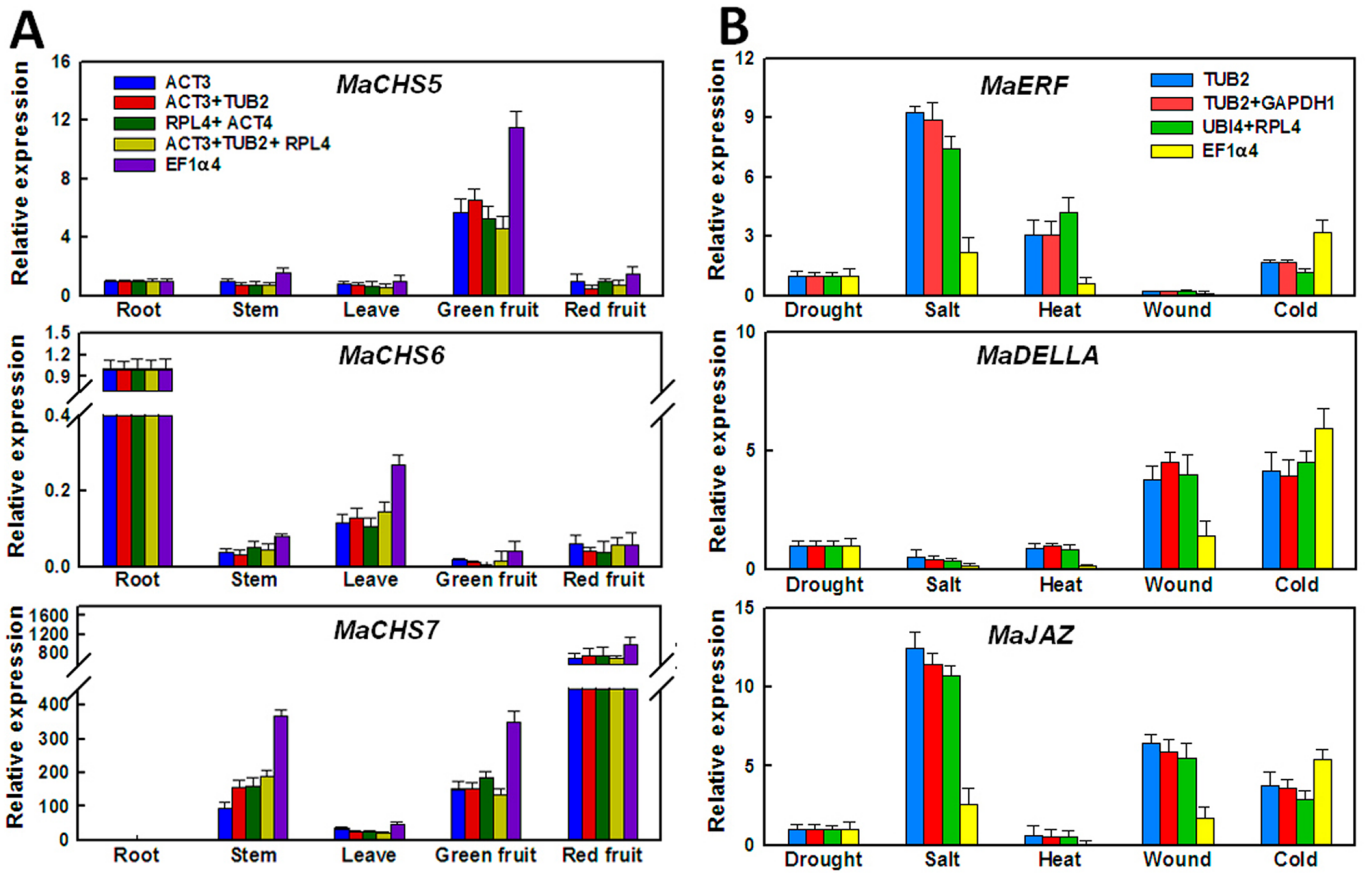
Relative quantification of several genes for different tissue samples and abiotic stress samples using selected reference genes including the most and the least stable reference genes for transcript normalization. (A) Relative expression of *MaCHS5*, *MaCHS6*, and *MaCHS7* for different tissue samples. (B) Relative expression of *MaERF*, *MaDELLA*, and *MaJAZ* for different abiotic stress samples. Standard error bars are indicated. Error bars indicate the standard error (n = 3).

To evaluate the reliability of the reference genes identified by geNorm and NormFinder, the relative expression patterns of three chalcone synthase genes (*MaCHS5*, *MaCHS6*, and *MaCHS7*) in different tissues samples and three plant hormone related genes (*MaERF*, *MaDELLA*, and *MaJAZ*) under different abiotic stress samples were compared by qRT-PCR using different combinations of reference genes for normalization (Fig 5). Mulberry is rich in secondary metabolism flavonoids, and chalcone synthase is one of the key enzymes involved in flavonoid biosynthesis [17]. *MaERF*, *MaDELLA*, and *MaJAZ* were the crucial transcription factors of plant hormone signaling (ethylene, gibberellins, and jasmonic acid), which play important roles in plant responses to a wide range of abiotic stresses [18].

We investigated the relative gene expression of three *MaCHS* genes in different mulberry tissues samples. Four different combinations of reference genes were used for the validation, including the most stable reference gene *ACTIN3*, two most stably pairs (*ACTIN3*/*TUB2* and *RPL4*/*ACT4*), and the least stably expressed gene *EF1α4* in the tissue samples subset (Fig 3 and Table 2). *MaCHS5* was highly expressed specifically in green fruit. Considering the expression of root samples as 1, the relative gene expression of *MaCHS5* in other four tissues samples was found to be unbiased when three most stable groups (*ACTIN3*, *ACTIN3*/*TUB2* and *RPL4*/*ACT4*) were used as reference genes for normalization. However, when the least stably expressed gene *EF1α4* was used as the reference gene, the relative gene expression of *MaCHS5* in three tissues (stem, leave, and green fruit) was about two times comparing with which used three most stable groups (*ACTIN3*, *ACTIN3*/*TUB2* and *RPL4*/*ACT4*) as reference genes (Fig 5A). *MaCHS6* and *MaCHS7* were specifically and highly expressed in root and red fruit, respectively. Similar to the *MaCHS5* expression trend, the relative expression of *MaCHS6* and *MaCHS7* were also markedly higher than other reference combinations for three tissues samples (stem, leave, and green fruit) when normalized by *EF1α4* (Fig 5A). Based on the reference genes selected for normalization, the expression of target genes can show greater variability. We noted that using the three best genes for normalization—*ACTIN3*, *TUB2*, and *RPL4*—produced similar results to those from the best gene pairs (Fig 5A). Thus, two reference genes were sufficient for normalization in different tissues samples, which further confirmed the conclusion of the pairwise variation analysis by geNorm.

Meanwhile, we surveyed relative transcript accumulation of three plant hormone related genes (*MaERF*, *MaDELLA*, and *MaJAZ*) under different abiotic stress samples using different combinations of reference genes. Four reference gene groups were used for the validation, including the most stable reference gene *TUB2*, two most stably pairs (*ACTIN3*/*TUB2* and *GADPH2*/*MDH2*), and one of the least stably expressed gene *EF1α4* in abiotic stress samples subset (Fig 3 and Table 2). Considering the expression of drought stress samples as 1, the expression profiles of the three target genes in other four abiotic stress samples showed similar trends when stable reference gene groups (*TUB2*, *TUB2*/*GAPDH1* and *UBI4*/*RPL4*) were used for normalization. When the least stably expressed gene *EF1α4* was used as an internal gene, the expression level of the target genes were showed observably lower than other reference combinations for three abiotic stress samples (salt, heat, and wound) (Fig 5B). These results validated the effects of reference genes for normalization in mulberry by the two algorithms.

## Discussion

qRT-PCR is the most commonly used method in gene expression studies because of its accuracy, sensitivity, and efficiency [19,20]. It is very important to select a suitable reference gene in qRT-PCR analysis to correct for any errors in RNA quantity and reverse transcription efficiency, and ultimately to determine the real expression of a target gene [21]. A suitable reference gene should maintain invariable expression across different experimental samples [22].

To our knowledge, this is the first published report to examine reference gene selection in mulberry, although some reference genes have recently been used in mulberry studies. *ACTIN3* (NCBI number of HQ163775) and *ACTIN4* (NCBI number of KT793030) has been used as reference genes in qRT-PCR analyses in mulberry [16,23,24]. *EF-1α* and *UBI*—as basic components of metabolic processes in organisms—have been used as control genes in expression analyses of the stress response in mulberry [25,26]. *RPL15* (Ribosomal protein L15) was used as a reference gene in the qRT-PCR analysis of different tissues and stress treatments in mulberry [27,28]. In this study, we found that the same reference gene *ACTIN3* (HQ163775) was one of the most stable reference genes among most subset samples, suggesting *ACTIN3* is a good housekeeping gene in mulberry. However, the stability of *ACTIN4* (KT793030) and *UBI* genes showed not very well (Table 2, Fig 3).

geNorm and NormFinder are two commonly used programs to evaluate and identify suitable reference genes [12,13]. In our study, the evaluation of the candidate reference genes from the two programs showed similar stability, although there were some differences between them (Table 2, Fig 3). The differences may be due to the distinct algorithms of geNorm and NormFinder [12,13]. The reference gene *TUB2* had stable expression patterns in all four sample subsets in this study (Table 2, Fig 3), and thus the reference gene can be considered good housekeeping genes in mulberry. In contrast, *TUB3* and *EF1α4* had varying levels of expression across the subsets (Table 2, Fig 3) and should not be used as reference genes. The three tubulin gene family members (*TUB2*, *TUB3*, and *TUB4*) varied in their stability under the same experimental conditions. This phenomenon is consistent with findings from other plants. For example, *GAPDH*s are stably expressed in several plant species—such as *Dioscorea opposita* and *Panax ginseng* [29,30]—but are not stably expressed in watermelon (*Citrullus lanatus*) and celery (*Apium graveolens*) [9,15].

We found that *TUB2*, *UBI4*, *ACTIN3* and *RPL4* were ranked as the most stable reference genes in the samples subsets examined from the geNorm and NormFinder evaluation (Table 2, Fig 3). *ACTIN* and *TUB*, which encode the basic skeletal components of plant organelles, are the most commonly used reference genes and are usually stably expressed in tissues and organs in plants [31,32]. *ACTIN7* is the most stably expressed gene in pear (*Pyrus L*.) and tung (*Vernicia fordii*) [33,34] but is not a suitable reference gene in *Arabidopsis thaliana* and *Rhododendron* [35,36]. *TUB*s are considered to be the best reference genes in water lily and soybean [37,38] but are not stably expressed in *Rhododendron* [35]. *GAPDH*, *EF-1α*, and *UBI* are involved in biochemical metabolic processes in organisms and are traditional reference genes in plants [31,32]. *EF1α* genes have stable expression patterns in cucumber, potato, and soybean plants [39–41]. However, *EF1-α* genes are not regarded as suitable reference genes in *Arabidopsis* and bamboo [11,42]. These results suggested that reference genes used in qRT-PCR should be validated in different species and under different conditions.

*TUB2*, *UBI4*, *ACTIN3* and *RPL4* were ranked as the most stable reference genes in the samples subsets.

In conclusion, this is the first study to perform a systematic evaluation of mulberry to validate candidate reference genes for qRT-PCR normalization in different plant tissues and under different stress conditions. Twenty candidate reference genes were assessed. *TUB2*, *UBI4*, *ACTIN3* and *RPL4* were ranked as the most stable reference genes in different tissues and under different stress conditions of mulberry. Moreover, *ACTIN3*/*TUB2*, *RPL4*/*ACT4*, *TUB2*/*GAPDH1* and UBI4/RPL4 were identified as the most stably expressed pairs in our study. These results provide useful guidelines for qRT-PCR data normalization of gene expression analysis in mulberry.

## Materials and methods

### Plant material and treatments

Cuttings (about 10 cm in length and 1 cm in diameter) of mulberry (*M*. *atropurpurea*) cultivar “Tang 10” (bred by our unit) were rooted and planted in plastic pots (50% sand, 50% peat moss). One cutting with a sprout was planted in each pot. Plants were cultivated in a greenhouse (day/night temperature, 25/20°C; relative humidity, 40–70%) at the Sericulture and Agri-Food Research Institute, Guangdong Academy of Agricultural Sciences, Guangzhou, China.

When they were ~90 days old, the seedlings were used for stress treatments and tissue collection. Tissue samples of leaves, stems, and roots were collected from 3-month-old plants growing under well-watered conditions. Tissue samples of green fruit and red fruit were collected from the fruiting stage of adult mulberry cultivar “Tang 10”. For drought treatment, plants were subjected to dry conditions with no irrigation for 10 days, at which point slight wilting occurred. Salt stress treatment consisted of watering with 200 mM NaCl for 5 days. For cold and heat shock treatments, plants were transferred to 4 °C and 42 °C, respectively, for 48 h. Wounding stress treatment consisted of mechanical wounding of a leaf three to five times with a sharp knife. The wounded area represented ~10% of the leaf surface. Samples were collected from the last fully expanded leaf 24 h after the conclusion of each stress treatment. The samples from the five tissues and five abiotic stress treatments were collected from three replicate plants, giving a total of 30 samples comprised of 15 tissue-specific samples and 15 abiotic stress treatment samples. Samples were immediately frozen in liquid nitrogen and stored at –80 °C until RNA extraction.

### RNA extraction and cDNA synthesis

Total RNA was isolated using the Mini-BEST Plant RNA Extraction kit (TaKaRa, Japan) with the addition of an on-column DNase I digestion according to the manufacturer’s instructions. RNA sample concentration and quality (RIN-RNA Integrity Number) were determined using the NanoDrop 2000 spectrophotometer (Thermo Fisher Scientific, US) and the Agilent 2100 bioanalyzer (Agilent Technologies, Palo Alto, Calif.) (S2 Fig). The quality of RNA samples was also evaluated by 1% agarose gel electrophoresis (S2 Fig).

First-strand cDNA was synthesized from 1 μg of total RNA in a total volume of 20 mL per reaction using the PrimeScript RT reagent kit with gDNA Eraser (TaKaRa, Japan) following the manufacturer’s protocol. The cDNA products were diluted 10-fold with nuclease-free water before being used in the qRT-PCR assays.

### Primer design and qRT-PCR

Specific primers for qRT-PCR were designed using the Primer 5.0 software (PE Applied Biosystems, Foster City, USA) with primer lengths of 21–26 bp and amplicon lengths of 100–250 bp (Table 1 and S1 File). To determine the efficiency of each primer pair, a mixture of cDNA from the 30 samples was used to perform qRT-PCR reactions (see below). Five-point standard curves of a fivefold dilution series (1:1, 1:5, 1:25, 1:125, and 1:625) of the pooled cDNA were used. Agarose gel electrophoresis and polyacrylamide gelelectrophoresis (PAGE) electrophoresis of the amplification products of each candidate reference gene were analyzed.

qRT-PCR was carried out on a LightCycler480 System (Roche) using the SYBR Premix Ex Taq II kit (TaKaRa, Japan). Reactions were performed using a total volume of 20 μL, which contained 1 μL of cDNA template (corresponding to 5 ng of the starting amount of RNA), 0.2 mM each primer, and 10 μL 2× SYBR Premix Ex Taq II. The PCR cycling conditions were as follows: 94 °C for 30 s, followed by 40 cycles of 94 °C for 10 s, 55–62 °C for 10 s, and 72 °C for 10 s in a 96-well reaction plate, and the annealing temperature was based on the Tm value of primers. The melting curve was recorded after 40 cycles to verify primer specificity by heating from 65 °C to 95 °C. Each qRT-PCR reaction was performed in triplicate (technical replicates) on samples from three individual plants (biological replicates).

### Statistical analysis

Two software programs, geNorm 3.5 and NormFinder 0.953, were used to assess the expression stability of each candidate reference gene according to their user manuals [12,13]. The Ct values were converted into relative expression values, which were calculated in Microsoft Excel 2007 using the highest expression value as the calibrator, and then they were imported into the geNorm and NormFinder software. All other statistical analyses were performed with Microsoft Excel 2007.

### Normalization of the verified genes

Specific primers for the three *MaCHS* genes (*MaCHS5*, *MaCHS6*, and *MaCHS7*) and three plant hormone related genes (*MaERF*, *MaDELLA*, and *MaJAZ*) for qRT-PCR were designed using the Primer 5.0 software (S3 Table). The relative expression of the target gene was calculated using the 2^−ΔΔCt^ method [43] with normalization using the genes indicated. Measurement of the verified genes expression was made with three biological replicates with three technical repeats per sample.

## Supporting information

S1 Fig Amplification of a specific PCR product for each gene tested with PAGE electrophoresis. (TIF)

S2 Fig Total RNA after agarose gel electrophoresis and RIN value for the 30 tested samples. (TIF)

S1 Table Sequences of 20 candidate reference genes. (DOCX)

S2 Table RPKM (Reads per kilobase of exon per million reads mapped) of these eighteen candidate reference genes selected from our transcriptome database in three mulberry varieties after infection with *R*. *solanacearum* for various time points. (DOCX)

S3 Table Information of *MaCHSs*, *MaERF*, *MaDELLA*, *and MaJAZ* genes. (DOCX)

S1 File Primers location and the alignment of their gene sequences with homologous genes from other plants. (PPTX)
