# Supplementary figures and images for: Identification and validation of reference genes for qRT-PCR analysis in mulberry (Morus alba L.)
Source: PLoS One. 2018 Mar 15;13(3):e0194129. doi: 10.1371/journal.pone.0194129 (PMC5854264; doi:10.1371/journal.pone.0194129)

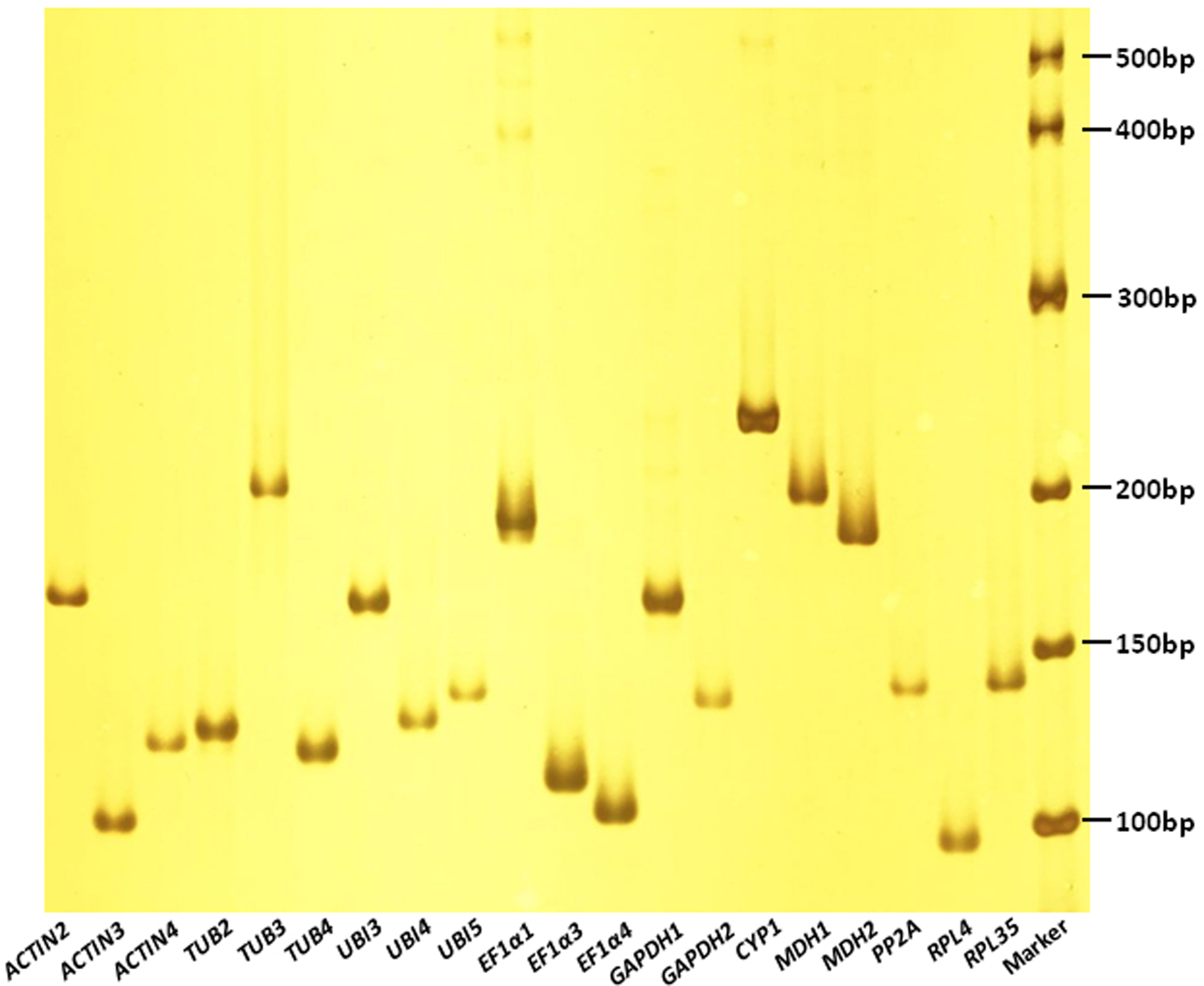

Supplement: S1 Fig — (TIF) [file pone.0194129.s001.tif]

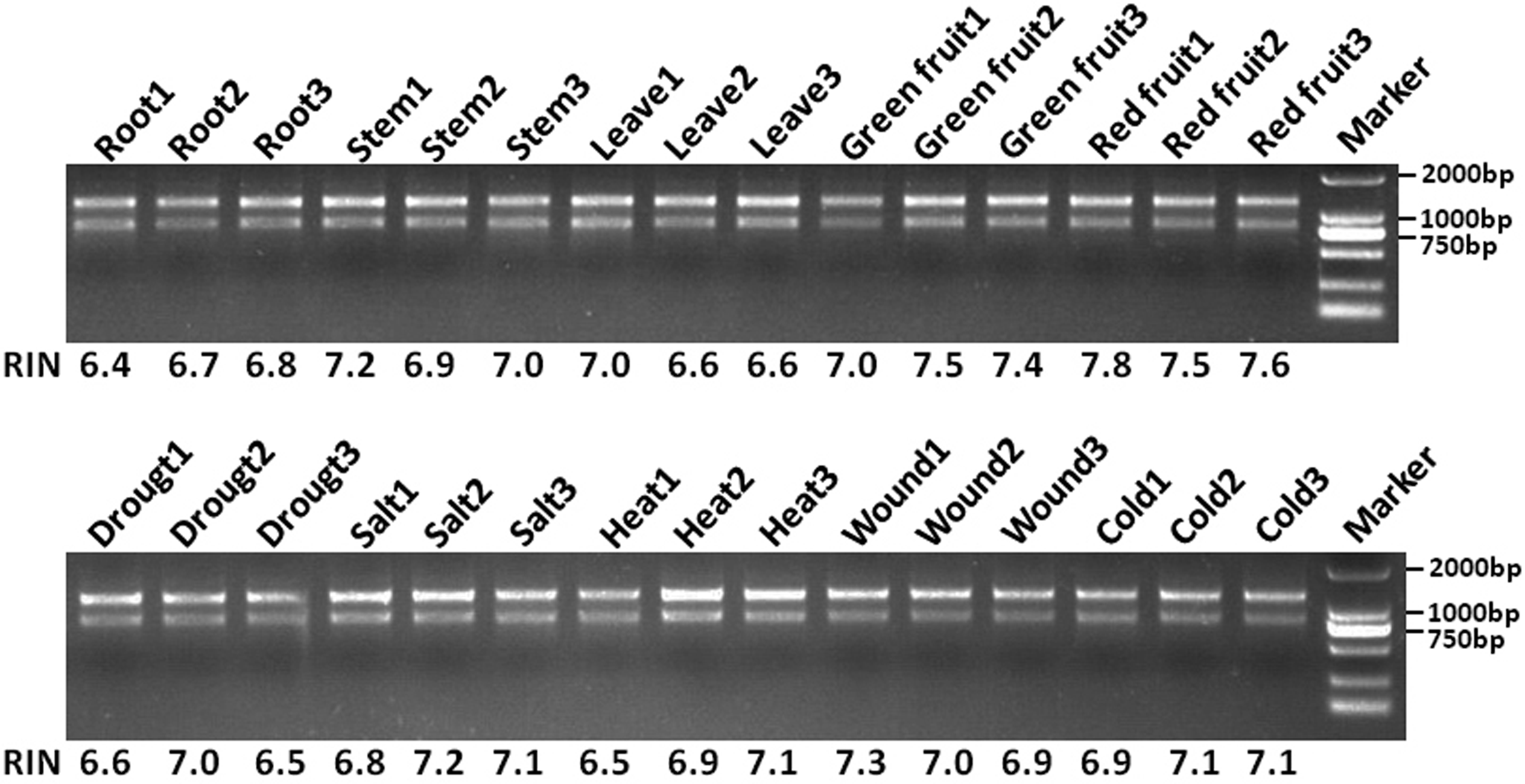

Supplement: S2 Fig — (TIF) [file pone.0194129.s002.tif]
