# Supplementary material for: Identification and validation of reference genes for qRT-PCR analysis in mulberry (Morus alba L.)
Source: PLoS One. 2018 Mar 15;13(3):e0194129. doi: 10.1371/journal.pone.0194129 (PMC5854264; doi:10.1371/journal.pone.0194129)
Supplement: S1 Table — (DOCX) [file pone.0194129.s003.docx]

S1 Table. Sequences of 20 candidate reference genes.

| **Gene name** | **Accession number/RNA-Seq**  **number** | **Gene sequence [(5′–3′)]** |
| --- | --- | --- |
| *ACTIN2* | HQ163776 | ATGGCAGATGGTGAGGAAATTCAGCCGCTTGTTTGCGATAATGGAACTGGAATGGTCAAGGCTGGGTTTGCTGGAGATGACGCTCCACGTGCGGTGTTCCCTAGCATTGTAGGTCGTCCCCGTCACACCGGTGTGATGGTTGGCATGGGCCAGAAAGACGCCTATGTTGGTGATGAGGCTCAATCCAAGCGTGGTATCTTAACTCTGAAATACCCGATTGAGCACGGTATTGTGAGCAACTGGGATGACATGGAGAAGATCTGGCATCACACTTTCTACAACGAGCTTCGTGTTGCCCCTGAAGAACACCCTGTTCTCCTCACTGAAGCTCCTCTCAACCCCAAAGCAAATCGTGAAAAAATGACCCAGATCATGTTTGAGACCTTCAACACCCCTGCTATGTATGTGGCCATTCAAGCCGTTCTTTCTCTATATGCCAGTGGCCGTACAACTGGTATTGTGTTGGACTCTGGGGATGGTGTCAGCCACACCGTTCCTATCTACGAGGGTTATGCCCTCCCTCATGCCATTCTTCGTCTTGACCTGGCTGGCCGAGATCTCACCGACCACTTGATGAAATTTCTTACAGAGCGTGGGTACTCCTTCACTACCACTGCTGAACGGGAAATTGTTAGGGACATGAAGGAAAAATTGGCTTACATTGCTCTTGACTTTGAGCAAGAGATGGAGACTGCCAAGACCAGCTCTGCAGTGGAGAAGAGCTATGAGTTGCCAGATGGACAGGTCATCACAATCGGTGCTGAGCGTTTCCGATGCCCAGAAGTTCTTTTCCAACCATCCATGATAGGCATGGAAGCTGCAGGCATTCATGAAACTACCTATAACTCAATCATGAAGTGCGATGTTGATATCAGGAAAGACCTTTACGGTAACATTGTCTTGTCTGGTGGTTCAACCATGTTCCCGGGCATTGCTGACAGAATGAGCAAGGAAATTTCTGCCCTTGCACCAAGCAGCATGAAGATCAAGGTGGTGGCGCCGCCCGAGAGGAAATACAGTGTCTGGATTGGAGGCTCTATCTTGGCATCACTTAGCACCTTCCAGCAGATGTGGATCGCAAAGTCGGAGTATGATGAGTCCGGGCCTTCAATTGTTCATAGGAAGTGCTTCTAA |
| *ACTIN3* | HQ163775 | GATAATGGTACTGGGATGGTCAAGGCTGGATTTGCCGGAGATGATGCTCCGAGGGCCGTGTTCCCCAGCATCGTCGGTCGCCCGCGCCACACCGGTGTGATGGTTGGTATGGGCCAAAAGGATGCCTATGTAGGGGATGAGGCTCAATCAAAAAGAGGTATTTTGACTCTCAAGTACCCAATTGAGCATGGTATTGTTAGCAATTGGGATGACATGGAGAAGATCTGGCATCACACCTTCTACAATGAGCTTCGTGTTGCCCCGGAGGAGCACCCGGTTCTCCTGACTGAGGCACCCCTCAACCCTAAGGCCAATCGCGAGAAAATGACTCAGATTATGTTTGAGACATTCAACACCCCGGCTATGTATGTCGCCATCCAGGCTGTCCTTTCCCTCTATGCCAGTGGGCGTACGACTGGTATCGTTCTTGACTCTGGAGATGGTGTGAGCCACACGGTTCCCATCTACGAAGGCTATGCCCTTCCACATGCCATCCTCCGTTTGGATCTTGCAGGGCGTGATCTCACAGACGCCCTGATGAAGATCTTGACGGAGCGTGGTTACTCTTTCACCACCACAGCCGAGCGGGAAATTGTGAGGGACATGAAGGAGAAGCTAGCCTACATCGCTCTTGGCTATGAGCAGGAGCTGGAGACGGCAAAGACCAGCTCAGCTGTGGAGAAAAGCTATGAGCTACCTGATGGACAGGTGATCACCATCGGTGCTGAGAGATTCCGATGTCCTGAAGTCCTCTTCCAACCATCGATGATCGGGATGGAAACTGCTGGTATCCATGAGACCACATACAACTCCATCATGAAGTGTGATGTTGATATCAGGAAGGACCTCTATGGCAACATTGTCCTCAGTGGGGGATCCACCATTTTCCCGGGTATTGCTGATCGGATGAGCAAGGAGATCACAGCGTTGGCTCCGAGCAGCATGAAGATAAAAGTCGTCGCACCCCCA |
| *ACTIN4* | Unigene23275 | AGGTTGTTGCTCCACCAGAGAGAAAGTACAGTGTCTGGATTGGAGGGTCAATTCTTGCTTCCCTCAGTACCTTCCAGCAGATGTGGATTTCCAAGGGCGAGTACGACGAGTCTGGTCCATCAATTGTCCACAGGAAGTGCTTCTAAGTTTTATAAGTGCTTTGATGGTGAGTTATTTTTACATTTAGTTGGGTTTTTTTTTTGTGTCATGTGTCATGTGAACTCAGTTTGGCTGAAGTGGACAAGTATTGAATTGAGAGAAGGACAAAATTGGGGTCATTGAATTCACGCTTGTCATCGTTCTTTGTGAGAAGCTTTTTTATCTGCAGTGGCCCTCTTGTATGTGGGCGGGAAATTGGTTCAAACTCCCTCGCAGCCCATCATCATGATGATCATCATATCTATGGTTCAACCCGTCTCTTTCTGTAGGATGTTTGTAGTTGGAGAGTGATTTGTGTTTTTTTTTTTTAATCTTTTATTTTTTTCTTTTCCCAATATTTTGACGGTTTTTTCAACCCAGAGAACATTAATGTTAATAGTTATTGTATCTGAAATTCAGTTAGTGTCAGTTTGCGGTCATAAACTTGCTATGAAATTATATTAAAATACTGGCTTGAAAGCTTGAGAGAATGTTTTCCTTTTTTCTTCTTTTTTTATAATT |
| *TUB2* | CL1595.Contig6 | GTTCCCTGGACAGCTGAACTCTGACCTCCGGAAACTGGCTGTTAACCTCATTCCTTTCCCTCGCCTGCACTTCTTTATGGTTGGATTTGCCCCACTAACTTCGAGAGGATCCCAGCAGTACCGTGCTCTCACCGTGCCTGAACTGACCCAGCAGATGTGGGATGCCAAAAACATGATGTGCGCAGCTGATCCACGCCATGGTCGTTACCTCACCGCTTCCGCCATGTTCCGTGGTAAGATGAGCACAAAAGAAGTCGATGAACAGATGATCAACGTCCAGAACAAGAACTCCTCATACTTTGTCGAGTGGATACCCAATAATGTGAAGTCTAGCGTGTGTGACATTCCACCCAAGGGTCTGAAGATGGCTTCGACTTTTATTGGAAACTCGACTTCAATTCAGGAGATGTTCAGGAGGGTCAGCGAGCAGTTCACGGCTATGTTCAGGCGCAAGGCTTTCTTGCACTGGTAC |
| *TUB3* | CL8311.Contig1 | CAAGGGTCACTACACTGAGGGAGCAGAGCTGATTGATTCGGTTCTTGACGTTGTTCGGAAGGAGGCGGAGAACTGTGACTGTCTTCAAGGTTTTCAAGTGTGCCACTCTCTTGGTGGAGGTACTGGTTCTGGAATGGGGACTTTGTTGATCTCGAAGATCAGGGAAGAGTATCCAGATCGGATGATGCTTACATTCTCTGTGTTCCCATCACCGAAGGTTTCAGATACAGTTGTTGAGCCCTACAATGCC |
| *TUB4* | CL2672.Contig2 | AAAAAAAACAAAAACAAGAACATAAACGAATTAATCAGACCAGAAATAATTGAATTCCTTCTCACCAAAGGTTCATCTAGCTACTCATCTTGACTGCTTGTTCTCTTGGTAGCGATCATCCTCTCTCTCTCTCTCTCTCTCTATTTCTTTCTCTCTCTTCCCAATTTTTTCCCTCAAAAGTTAGACTCGCGCAAACCCTACTTTGATCTCTCCATTTCCATCGGAAGGCAGAGACGTATAGAGAGATATTTTCTTGTTCGGTTTTCCTTTTGACCAAACAGACCAAGAAGAGGTAGAAAAATAAAGAGATGAGAGAAATTATAAGCATTCACATTGGGCAGGCTGGCATTCAGGTGGGAAATTCTTGCTGGGAGCTTTACTGCCTTGAGCATGGCATTCAGCCCGATGGGATGATGCCCAGTGACACTACCGTAGGTGTCGCACACGATGCTTTCAACACGTTCTTCAGCGAGACGGGCTCGGGCAAGCATGTGCCTAGGGCCATTTTCGTCGATTTGGAACCTACTGTTATCGACGAGGTCAGAACTGGTGGATACCGCCAACTCTTCCACCCCGAGCAGCTCATTTCTGGCAAGGAGGATGCTGCTAACAATTTCGCGAGGGGGCACTACACAGTTGGAAAGGAAATTGTCGATCTCTGCCTGGACCGTGTAAGGAAATTGGCTGATAACTGCACCGGACTGCAAGGGTTTTTGGTGTTCAGTGCTGTTGGTGGTGGTACTGGCTCTGGTTTGGGGTCCTTGCTACTAGAACGCTTGTCGGTGGATTATGGGAAGAAGTCGAAGCTTGGCTTCACCATCTACCCTTCACCCCAGGTTTCAACTGCGGTGGTTGAGCCTTATAACAGCGTTCTCTCCACACATTCCCTTCTTGAACACACAGATGTGGCTGTGCTGTTGGACAATGAAGCTATTTACGACATTTGCCGGAGGTCCCTAGACATTGAAAGGCCAACATACACCAACTTGAACCGCTTGATATCCCAGATCATATCATCCTTAACAACCTCGCTGAGGTTTGATGGAGCCATCAATGTTGACATCACAGAGTTCCAGACGAACCTTGTGCCATATCCCCGTATTCATTTCATGCTTTCCTCGTATGCCCCCGTCATCTCGGCTGAGAAAGCATACCATGAACAACTATCAGTCCCTGAGATCACAAATGCCGTGTTTGAACCTTCAAGCATGATGGCAAAATGTGATCCCAGGCATGGGAAATACATGGCCTGCTGTTTGATGTACAGAGGAGATGTTGTTCCCAAGGATGTCAATGCTGCTGTTGGCACTATCAAAACGAAAAGGACTGTTCAGTTTGTTGACTGGTGCCCAACTGGCTTCAAGTGTGGCATAAACTACCAGCCTCCAACAGTCGTACCCGGTGGTGATCTTGCCAAGGTGCAGCGAGCGGTTTGCATGATCAGCAACAACACAGCAGTGGCTGAGGTCTTCTCGCGCATCGACCACAAGTTTGATCTCATGTATTCCAAGAGGGCGTTCGTCCACTGGTATGTCGGTGAAGGCATGGAAGAAGGTGAGTTCTCAGAAGCTCGTGAGGATCTTGCCGCCCTTGAGAAGGATTACGAGGAAGTTGGCGCTGAAGGTGCAGATGACGAAGAGGAAGGTGAAGACTACTAATGATGTTGTGCAAGTGCACTTTACCTTGAATATGAAGAATATATGAAGATTTTCTCTTCACCCTTTTCCTTTATTTCGCTTTTGCTACTGTTTGTTTTGTGCAGAACAATCCATTTTATCCACCGTTTTTCCATATGGAATTGTCAAAAATATGTCCTTAATTGAATATTCTCGTTTTGCTTTTTGGTATCAATGAAGTGATTCTTTAGAATCATGGCTTGAACATAAAGTTCTGTAGGAAATATTACAATTTGTTTCCTACTTGAGGAAAAG |
| *UBI3* | Unigene18627 | CACTCGAATGAACAGAAATTTCTAGAAGCGAGGATTTTGAAGTAACCCGCTTAATTTCCTTATTTATGAGTCTTTTGGCCCTGGAAGACTCGAGTAAAAGACAAAATCACGAATTTGGGAATTACGATTTGGGGAATTAGGGTTTGCGATCAAGAAGAAAGAAGGAGAAGATGATCGAGGTGGTGTTGAACGATCGGCTGGGGAAGAAGGTGAGGGTGAAGTGCAACGATGACGACACCATTGGAGACCTGAAGAAGCTCGTGGCGGCTCAGACAGGCACGAGAGCCGACAAGATTCGGATTCAGAAGTGGTACAACATCTACAAGGACCACATCACTCTCAAGGACTACGAAATCCATGACGGGATGGGCCTCGAGCTCTACTACAACTGATTTCGCCCTTCTTCCGTACGTATGTTCTATACGCTAGTGGAGCTTGGGAGATGGATGCCATTTCTAAATCTGAACTTGTCCGAATGAACTTGCTGTTATGATGTTGGTTTACCTAAAAATGTCCTGTACTTATCTTATGCTGGTTGGCACTTCATTACTTGTCTAGATGTGCCAATCCAATTTGCTTGCGAGTCTTTAAGTGTCTGACTTATATTATTTAAGTTTCAACTCTTTGCCGTGTGTCTTGTTATTCAATGTGATTGTGGCGTTTTATGGTTTTTGCACTTCGACTCTGAAACCCATTTTATAGCCATACATCTTAAAAATATTCGCATGTCAAACCTTTGACGTTAGATCATTAAAATGCTTTCAGTTTATATTGAAGACTGGAGATGCTGTTGCCATGTTAATCAGGGGTTTGATAT |
| *UBI4* | Unigene5350 | TCTCTCTCTCTCTCTCTCTCTCTAACCCCGAGAAATCTCTCACTTCGATCGGATCTCCTTAAACCCACCTGAGGAAACCCCCCAAACCTCATCATCACCATCATCATCATCATCCATGACGCTTGGCTCAGGCGGATCGAGTGTCGTGGTCCCTCGGAACTTTAGACTGCTGGAGGAACTGGAACGGGGTGAAAAAGGCATTGGAGATGGCACTGTCAGCTATGGAATGGACGACGGAGATGACATTTACATGCGCTCATGGACTGGCACCATAATTGGTCCTCACAATACTGTGCATGAAGGTCGAATTTATCAGCTGAAGCTGTTCTGTGATAAGGACTACCCAGAGAAGCCACCAAGTGTTCGTTTTCATTCACGTATCAACATGACCTGTGTCAACCATGAATCTGGGATGGTGGAGCCCAAGAAGTTTGGACTTCTTGCGAATTGGCAGCGCGAGTACACCATGGAAGACATACTGACACAGCTGAAGAAGGAGATGGCTGCCCCGCACAACCGGAAGTTGGTTCAGCCTCCAGAAGGTACCTACTTTTAGCTTTCAAGAAGATCTTACACAATATCATGGATCATCTTGCATTTGCAATATGCAATATATAGTCTGTAATGCTGTGACAATCCTGTTTAGAACATACAGGGGAATGCCCCCCAAAAGCTTCTTCTTCTACCACCTCTCCATGGCTTCCTTGTTGCTTTGTACTCCTCTTTTGTCGATTTACATTTCAGCGATGCTGTTTAATTAATGGGATTTTATGTGTGTCAAATATTAAAACCTGTTAACTAGTATCCATTCTAGCTTTATCAGTGTACTAAAATCACTTCCCTTTCTTTTGCCCCATTGCTCTCTTTCTTTCCTTGTTAATTCAAGGATAAAAAATGTTAATGGTACTTATCCAAAAAAAAAAAAAAGTTGCGAATTTGGAAAGTACTTTGGAGTCGTGAAAAGTGATGTGGATGGCGCGGATTTGATCTTGAATTGTATGAAAAAAAAAAAAAAAGGCTCTGCCGTGGTTAATCCGTC |
| *UBI5* | CL1942.Contig2 | AAGCTCCCATGTGCACGGTGCATTCCTTTCCACACAATATTGATCACTGCTTGACATGGGCCCGATCTGAATTTGAGGGTTTGCTTGAGAAGACACCGACTGAAGTAAACACATACTTGTCCAATCCAAGTGAGTATGCGACGTCAATGAGAAATGCTGGTGATGCCCAGGCCAGAGATACCTTGGATCGTGTTCTTGAGTGTCTTGACAGAGAAAAATGCGAGAGTTTCCAAGATTGTATTTCCTGGGCTCGCCTAAAGTTTGAAGATTACTATGCTAACCGTGTGAAGCAGTTGATTTTTACTTTTCCTGAAGATGCTGCAACCAGTACTGGGGCTCCATTCTGGTCAGCTCCTAAGCGATTCCCGCATCCTCTGCAGTTCTCAGCTGCCGATCCTGGTCACCTCCATTTTGTTATGGCAGCATCAATACTAAGAGCGGAGACATTTGGTATTCCAATTCCTGACTGGGTTAAAAACCCTAAGAAGTTGGCTGAAGCTGTAGATAGAGTGATAGTCCCTGAATTTCAGCCCAAGGAAGGTGTCAAAATTGAGACTGATGAGAAGGCCACCAATGTTAGTTCTGCTGCGTCTGTCGATGATTCACTGATAATTAATGAATTAATCACGAAGTTGGAGCACAGTCGAGCAAGCTTGGCACCAGGGTTTAAGATGAAACCAATTCAGTTTGAGAAGGACGATGATACCAACTACCATATGGACATGATTGCTGGTCTTGCCAACATGAGGGCCAGGAATTATAGTATTCCCGAGGTTGACAAGCTGAAGGCCAAGTTTATTGCTGGAAGGATTATCCCTGCTATTGCGACATCCACTGCTATGGCGACGGGTCTCGTCTGCTTAGAGCTATATAAGGTTTTGGATGGTGGCCACAAACTTGAGGACTACCGAAACACATTTGCAAATCTAGCACTGCCTTTGTTTTCAATGGCTGAGCCAGTTCCGCCTAAGGTCATCAAGCACCGCGAAATGAAGTGGACTGTTTGGGATAGGTGGATCGTGAAGGACAATCCAACTCTGAGGGAACTTCTTGAGTGGCTAAAAAATAAAGGGCTAAATGCTTACAGCATCTCCTGTGGAAGTTGTCTGCTGTATAATAGTATGTTCACGAGACACAAGGATCGAATGGACAAGAAGGTGGTAGATTTGGCTAGGGATGTTGCGAAGGTAGAGTTGCCTGCATACCGCCGACACTTGGACGTGGTTGTGGCATGTGAGGACGACGATGACAACGATATTGATATCCCTTTAGTATCAATTTACTTCCGTTAAGTCGTATTTCTAAATCCACCGATGCTGTCGGAATGTGATTTTAGGATGAGAGCCCTTAAATAATTCTTCTGACTTGCTTTTAAATGAATTATCTCGGCACACATGTTGAACATTTTTCGAAGTTATGTTTCATATATAAATCTGAAAGACCTCTTTAGTTGGATTTGTGG |
| *EF1α1* | CL4198.Contig2 | CTAGATTTTTTCCCGAGCTATAAATATAGGACAAAGCCATTTCCCAAACCCTATCGCTCAGTTTGTGCTTTCGCTGCTGCTCTAGGGTTTTAACTTTTCCTCTCTGAGCAGCCGCTTCAAATCTTCTCTCTTTTAAGGTTTTTGATTCATCATGGGCAAGGAAAAGATCCACATCAACATTGTGGTCATTGGCCACGTTGACTCTGGAAAGTCGACCACCACCGGTCACCTCATCTACAAGCTTGGAGGTATTGACAAGCGTGTGATTGAGAGGTTTGAGAAGGAAGCTGCTGAGATGAACAAGCGTTCATTCAAGTATGCCTGGGTGCTTGACAAGCTCAAGGCCGAGCGTGAGCGTGGTATCACCATCGACATTGCCCTGTGGAAGTTCGAGACCACCAAGTACTACTGCACTGTCATTGATGCTCCTGGCCATCGTGACTTTATTAAGAACATGATCACTGGTACCTCGCAGGCTGACTGTGCCGTTCTCATCATTGACTCAACCACTGGTGGTTTTGAAGCTGGTATTTCCAAGGACGGCCAGACCCGTGAGCACGCTCTCCTTGCTTTCACCCTTGGTGTTAAGCAGATGATATGTTGCTGTAACAAGATGGATGCCACCACTCCCAAATATTCGAAGGCTAGGTACGATGAAATTGTGAAGGAAGTCTCATCTTACTTGAAGAAGGTCGGATACAACCCTGACAAAATCCCATTCGTTCCCATCTCTGGATTCGAAGGTGACAACATGATTGAGAGGTCCACCAACTTGGACTGGTACAAGGGTCCCACCCTCCTCGATGCTCTTGACCAGATTCTGGAGCCCAAAAGACC |
| *EF1α3* | CL272.Contig1 | ACCAGATTCTGGAGCCCAAAAGACCAACAGACAAGCCTCTCCGTCTCCCACTCCAGGATGTTTACAAGATCGGTGGTATTGGAACTGTCCCAGTGGGTCGTGTTGAGACTGGTGTCCTCAAGCCCGGTATGGTTGTGACCTTTGGACCAAGTGGACTAACCACTGAAGTTAAGTCTGTTGAGATGCACCACGAGGCTCTCCAGGAGGCTCTCCCGGGTGACAACGTTGGGTTCAACGTTAAGAATGTGGCCGTCAAGGATCTTAAGCGTGGTTATGTCGCCTCCAACTCCAAGGATGACCCTACCAAGGAGGCTGCCAACTTTACATCCCAGGTTATCATCATGAACCACCCTGGCCAGATTGGCAATGGCTATGCCCCAGTTCTTGACTGCCACACCTCCCACATTGCTGTCAAGTTCGCTGAGCTTCTTACCAAGATTGACCGTAGGTCTGGAAAGGAGCTCGAGAAGGAGCCCAAGTTCTTGAAGAACGGTGATGCCGGTATGGTGAAGATGATTCCCACTAAGCCCATGGTGGTGGAGACCTTCTCCGAGTACCCCCCACTTGGCCGTTTTGCTGTGCGTGACATGCGCCAGACTGTTGCTGTTGGTGTCATAAAGAGCGTTGAGAAGAAGGACCCAAGTGGAGCTAAGGTCACCAAGTCCGCTGCCAAGAAGGGAGGGAAGTGACTGGCTTTTTGCCACGAGATTTTCTTCTTCATTGGTGGATTATGGAGCTACTTTTTGTGTTTTTATTATGATCGTGCTAAATACTTGTCTTATTTTGATCGTGTTGTCTTGTGCTCGCTGACCTTTCTTTGCGCTCAGAACTGGGTGCTCGATAGGCGGTGGCATCTTCTTTTGTGAAGATGTTTTTTTCCGAACAGTTTTCCATTAGGCGTTTTATCATTTGCTCTTGTTGAACTCATTAAAGTTCTGGAAGTACTTTACTAGCTTACATTTTGTTCTTAATCTATATCTTGTTGCTCTGAAAAAAAAAAAAAAAAAAAA |
| *EF1α4* | CL155.Contig3 | CAAGAACATGATCACCGGCGCCGCTCAGATGGATGGCGCGATTCTCGTCGTCTCCGGGGCCGACGGCCCCATGCCGCAGACGAAGGAGCACATCTTGCTCGCCAAGCAGGTCGGCGTCCCGAACGTGGTCGTCTTCTTGAACAAGCAGGACCAGGTTGACGACGAGGAGCTTCTCCAACTGGTTGAATTGGAGGTCAGAGATCTTCTCACCTCCTACGAATTTCCCGGCGACGAGGTTCCAATGGTCTCTGGCTCCGCATTGTTGGCTCTAGAGGCTCTAATGGCGAACCCCAAAATCAAGCGGGGAGAGAATCAGTGGGTTGATAAAATATACGATTTGATGGATGAAGTTGACAATTACATTCCAATTCCCCAGAGGCAAACCGATCTGCCGTTCCTCTTGGCGATTGAGGATGTTTTTTCGATCACCGGCCGCGGGACGGTGGCGACAGGGCGTGTTGAGAGAGGAACTGTCAAGGTTGGAGACACTGTGGATATAGTTGGGCTTGGAGAGACTAGGAGCACCACAGTTACTGGGGTTGAGATGTTTCAGAAGATCCTTGATGAGGCTCTTGCTGGTGACAATGTGGGGGTTTTGCTTAGAGGAATTCAGAAGACTGATATTCGGAGAGGGATGGTTATTGCCAAGCCTGGCACCATCACCCCGCACACAAAATTCGAGGCCATTGTGTATGTTTTGACGAAGGAAGAAGGCGGGAGGAGCTCGCCGTTCTTTGCAGGTTACCGGCCGCAGTTTTACATGAGGACGACCGATGTTACCGGGAAGGTTTCTAAGGTTATGAATGACAAGGATGAGGAGTCCAAGATGGTTATGCCTGGCGACCGGGTTAAGATGGTTGTGGAGCTTATAATGCCGATTGCTTGTGAGCAAGGGATGAGGTTTGCCATTAGAGAAGGTGGGAAGACTGTTGGAGCTGGTGTCATTCAGTCAATCATCGAATGATCTATTTAGTTATGTTAGTTTTTCTGTTTGTTGAATTATCTTTGTGTTCTCTTTGTGGGACTTTAGGTGTTACATTTTGTTGATAGGTTAATGTAGAATGAATAATGAAACATGTTTGTTTCTCATATCTGTATGCTGCTAGTGATGTTAATTGTTACAATTTTGAAGCTAGGCGCGATTATAGGTTTGTACTTGCCTGTCAAATGGGTTTGAATATAAATGTAATTTGCATTGCCTCCGGATTTTCACAATGTAGAGTTCTCAAATTAC |
| *GAPDH1* | CL3772.Contig5 | ATCCCTAACATTGGCATTTCCTCGTTAGCTCTCACTATCTCACTCTCACTCTCTCTCTCTCTCTCTCTACTCTTTCGCTCGAAACTTCTCGCCGTCTCTACTTTCTCTCTCTTCTTTGATCATGGGGAAAATCAAGATCGGAATCAACGGTTTTGGAAGAATCGGTAGGTTGGTGGCTAGAGTTGCTTTGCAGAGAGACGATGTTGAACTCGTTGCTGTTAACGATCCCTTCATCACCACCGATTACATGACCTACATGTTCAAGTACGACAGTGTTCACGGTCAATGGAAGCATTTTGACCTCAAGGTCAAGGACGAAAAGACCCTTCTCTTTGGAGGGAAGGCCGTCACCGTTCATGGCATCAGGAACCCAGAAGAGATCCCTTGGGGTGAGAGTGGAGCGGATTTCGTTGTGGAGTCCACCGGAGTTTTCACTGACAAGGACAAGGCTGCTGCCCATTTGAAGGGAGGTGCAAAGAAGGTCATCATCTCAGCTCCTAGCAAGGATGCTCCCATGTTTGTTGTGGGTGTCAACGAACATGAGTACAAGCCAGAGCTCAATGTTGTTTCCAATGCTAGTTGCACTACCAACTGTCTTGCTCCCCTGGCCAAGGTCATTAATGATAAATTTGGAATTGTCGAGGGTTTGATGACCACTGTGCACTCGATCACTGCCACACAAAAAACTGTTGATGGACCCTCAGCGAAGGACTGGAGAGGTGGAAGAGCTGCTTCATTCAACATCATTCCTAGCAGTACCGGTGCTGCGAAGGCTGTTGGGAAAGTCCTGCCGTCTTTGAACGGAAAGTTGACTGGAATGGCTTTCCGTGTTCCCACTGTTGATGTTTCAGTGGTCGACCTCACAGTAAGGCTGGAGAAGGCGGCTACCTATGAGCAGATAAAGGCTGCAATCAAGGAGGAGTCCGAGGGAAAACTGAAAGGCATCTTGGGTTACACTGAGGAGGACGTGGTTTCCACTGATTTTGTTGGTGACAGCAGGTCAAGTATCTTCGACGCAAAGGCCGGAATTGCTTTGAATGAGAAGTTTGTTAAGCTCGTTTCGTGGTACGACAACGAGTGGGGTTACAGCTCCCGTGTGGTTGACTTGATTGTCCACATCGCTAAAACCCAGTGAAAATGGCTAAGACTGGCTTCTTCGGTATGCTATAATGCTACTAATAGCGTCCCATCGTATTGGCTGATTTTATTCAGTCTTCTGCTTCATATCGACAATAAGAGATGTGCCACAAACGACTTTGGTATTTGTGCCAAGTCTTTTGTACTTTAGATCTTTGAGGTCTCGTTGTAATGGGGGAGAATACCCTATGTTTTAGTTTTCAAGAGTATTGAACCTGTTTGACGGTATATGTGTTTTGCTTAAATTTTCTCTCTCTCTCTCTCTCTCTCT |
| *GAPDH2* | CL131.Contig2 | GTCTTAGAAGTGTTGCTCTTGTACTCCAAGCCCAGCAGCCGCCCTCCGGCCTCCACCTGTCAACAAACACCCTTATGCTCTCTGACTTTCCAACACTAAACAAACAATTTAGAGTTGGCTCAAACCGAGAAAGAGTAAGATCAATTCATAGGCAAAATATGTCCGGAAGTGAAGCTTTTGCAGAGATCATAGATGGCGATGTGTACAAGTACTATGCAGATGGTGAGTGGAAGAAATCCGCTTCTGGAAAATTCGTGCCTATTATCAACCCCACCACCAGAAACACCCAATTCAAAGTGCAAGCTTGTACTCAAGAGGAGGTGAACAAACTAGTAGAAACGGCGAAATTAGCACAAAAATCATGGGCAAAAACTCCTTTATGGAAAAGAGCAGAGTTACTTCACAAAGCAGCTGCAATACTGAAACAGCACAAAGCTCCGATCGCAGAGTGTCTCGTTAAGGAGATAGCAAAACCAGCGAAAGACGCTGCGACTGAGGTTGTCAGGTCAGGGGATTTGGTGTCGTATTGTGCTGAAGAAGGAGTGAGAATTCTCGGAGAAGGGAAGTTTCTGGTATCTGATAGCTTTCCGGGCAATGAGAGGACCAAATACTGCCTCACTTCAAAGATTCCACTGGGTGTTGTTTTAGCTATCCCACCCTTTAACTATCCTGTCAATCTTGCTGTCTCAAAGATTGCTCCTGCCCTTATTGCGGGAAACTCCATTGTGCTCAAGCCCCCGACTCAGGGTGCTGTTGCTGCACTTCACATGGTGCATTGCTTTCATTTGGCCGGTTTTCCAAGAGGCCTTGTGAGTTGTGTCACCGGGAAAGGTTCTGAGATTGGCGACTTCCTCACTATGCATCCAGGGGTCAGCTGTATAAGCTTCACAGGTGGGGACACCGGAATAGCTATCTCAAGAAAGGCTAGCATGATTCCTCTTCAGATGGAGCTAGGTGGAAAGGATGCGTGCATTATTCTTGAGGATGCTGATTTGGATTTAGCAGCGGTTAGCATCGTAAAAGGAGGCTTTTCTTATAGTGGTCAAAGGTGCACAGCCGTAAAGGTTGTGTTGGTCATGGCATCGGTAGCTGATACTCTTGTCGAGAAAGTCAATTCTAAGTTGGCAAAATTAACTGTTGGGCCACCGGAGGACGACTGTGATATTACTCCAGTTGTCACGGAGTCTTCCGCTAACTTCATCGAAGGGTTGGTCATGGATGCCAAACAGAAAGGAGCCACATTCTGTCAAGAATACAAGAGGGAAGGCAACCTCATATGGCCATTGTTGTTGGACAATGTTAGACCAGATATGCGAATAGCATGGGAAGAGCCATTTGGTCCGGTTTTGCCGCTTATTCGTATTAACTCTGTTGATGAAGGAATCCATCACTGTAATGCTAGCAATTTCGGCCTTCAGGGCTGTGTGTTCACTAAAGACATAAACAAGGCAATCTTAATCAGCGACGCGATGGAGACCGGTACTGTGCAGATCAACTCGGCACCAGCTCGCGGACCGGATCATTTCCCTTTCCAGGGTTTGAAGGACAGTGGCATTGGTTCTCAAGGGATTACTAACAGCATTAACATGATGACAAAGATAAAGAGTACTGTAATCAACTTGCCGTCCCCTTCATACACAATGGGATGATGCGCTTTGGAATGAGAAGGCAATACAATATATATATATATAGATTGTACTATGGTCTATGTATCGGTATATTCACATGTTATGTCTTGCCATATATGTGATGATAATTTTACTTTTCTAAGGTTATGGAACAATCTGAATTTTTGTTATGCTGCACTTGAACAATCTGAAAATTTTTAACTATTCATAATATATATTTCTTCTCCGAT |
| *CYP1* | Unigene15318 | CCAAACCCAATCACGGTTACCATAACTCCGTTGGATCACGTCACGTGTTTGAATCGGACGGTTGAAAATAAAACTTCCATAGATAATTGCAGCGATGACCTCTATATAACCTCCTCGTCCACGACTTCGCCTCAAGCCCTAGCAAGTCTGCTCTTTAGCCATTTCTCATTTTCAGTGACAAAAAAACCTCGAAAGTTCCTCATCAATGGCGGCAAACCCTAAGGTCTTCTTCGACATCGCGATCGGCAACACACCTGCCGGCCGGATCGTGATGGAGCTCTACGCCGACGTCACCCCCCGCACGGCCGAGAACTTCCGCGCCCTCTGCACCGGAGAGAAGGGAGTCGGACGGAGCGGAAAGCCTCTCCACTACAAGGGATCGTCCTTCCACCGTGTGATCCCCGGGTTCATGTGCCAGGGCGGCGACTTCACCGCCGGAAACGGCACTGGCGGCGAGTCGATCTACGGCTCCAAGTTCGCTGACGAGAACTTCATCAAGAAGCACACCGGCCCTGGTATCCTCTCCATGGCGAATGCTGGCCCCGGGACCAACGGATCTCAGTTCTTCATCTGCACGGCCAAGACCGAGTGGCTCGACGGCAAGCACGTGGTGTTCGGGAAGGTCATCGAGGGCTACGACGTCGTGAAAGCGGCGGAGAAGGTGGGATCCAGCTCCGGCAGGGTCTCTAAGCCCGTGGTGATCGCCGACTGTGGCCAACTCTCTTAGATTTATATATATCTGTTTGGAAAAAGTCTTTCTTCTGCTAATAGGTGTCTAATTTTGGTGTTTTAGTTATGTTATTGTCTCTACTCTTTTGGGATGTGACAGTGGCTATATCGAATGGTTGGATACTTTGTCTCTTTATTATTGCCTCTGTTAGCTTTGTTTGCTAGTTTCTGAGACTCTTATTATGATCCAAACAAGCTATCTTTTAAATAAAAAATAGGCCCTTTTCTGAAAAAAAAAAAAAAAAAAAAAAGAGAGAGAGAGAGAGAGAGAGATGGGAATTTCAGACTTTCTTTTTCTTGTTGGTTGAAAGCGTAAACCAAGTGGAG |
| *MDH1* | Unigene16243 | CGGGTGCCAAGTGATGAAACCACTCCCTTCGAGACTCTCAAACCCAAAACCCAAACCCAAACCCAAACCCTCTCTCTCTCTCTCTTCGAAAATTCCGAAAATGAGGTCTTCCATGCTTAGATCTGTCAAGTCGGCCATAGCCAGAAGCTCATCGGCAGCCGGAGCACAAATCCAGCGTCGTGGCTACTCCGTTCACTCCGGGCCGGAACGGAAAGTTGCTGTTCTCGGCGCCGCTGGAGGCATCGGACAGCCCCTCGCCCTCCTGATGAAGCTCAACCCCCTCGTCTCCCACCTCTCCCTCTACGATATCGCCGCCACCCCCGGCGTTGCCGCCGATGTCAGCCACATCAACACCAGATCTGAGGTTAAAGGATATGCGGGTGAGGAGAATATTCGGGAGGCTTTGGAGGGAGCCGATGTGGTGATCATTCCCGCTGGAGTGCCTAGAAAGCCCGGCATGACCCGTGATGATCTCTTCAACATCAATGCCGGCATCGTCAAGTCTCTCTGCACTGCGATCGCCAAGTACTGCCCACATGCTCTTGTGAATATGATCAGCAACCCTGTCAACTCAACGGTGCCAATTGCCTCTGAGGTTTTCAAGAAAGCGGGGACATATGACGAGAAGAGGTTGTTCGGTGTTACTACTCTTGATGTGGTTCGGGCCAAGACTTTCTACGCTGGGAAAGCCAAGGTCCCGGTTGCTGAGGTTAACGTGCCTGTTGTTGGTGGCCATGCTGGCATAACTATCCTTCCACTGTTTTCTCAAGCCACACCGCAAGCTAATTTGTCAGCTGAAGACATCACGGCTCTTACAAAGAGAACGCAAGATGGAGGAACGGAAGTTGTGGAGGCTAAGGCTGGAAAGGGATCAGCAACACTGTCCATGGCCTATGCAGGAGCTCTTTTTGCCGATGCATGTCTGAAGGGACTTAATGGTGTTCCTGATGTTGTGGAATGTTCTTTTGTGCAATCAAATGTCACTGAACTTCCATTCTTTGCTTCTAAGGTTCGTCTCGGAAAGAATGGTGTAGAGGAGGTTTTGGGGCTGGGTTCTCTCTCAGATTACGAAAAGGAAGGATTGGAGAAGTTGAAGCCCGAGCTGAAAGCTTCGATTGAGAAGGGTATAAAGTTTGCCAATGCGAACTAGATGAAACCATCCAAATTTTTGCAGTTTTAAGCAATTGTTGTATTATTGCTGCTACCAGCACCGGCTTTTTTTGCTGTTTCTTAGAGTAGGCCATACATCATAAACTGCAAATTCCGTTAATAATTCCCAGTGTTGTGTTTCTGGGAGGGACTTGAGAACTTGGATGCTAATATTTCTGGATAATTCGCAATTTTTTTGCTGCTTGAAAATGCATGCTCCCAATAAAATAGGATTTTACTGCGTGGTTTTTGAGGAGTCGGTATTTAGATCTTGATTTTCTCTGGGTTTGCTTAAGTGTTTGCTTTTGCATTTGCGGTTGGATTTTGGAGGTGGTCTTTGTAGTATTAGAATCTTGTGGACGATAGTCGAAGCCTCTTAAAAAAATCTTTTTTTTTTTTTTTTTTTTCTCTCCTTAGGATTTAAGAATCAAGCTCAACTTCTCCTACGATTGAGAGAAGAAAATATGAAATTCAGACATGTTTTAGTTTGGGCCTCTGTTTGAGGAAAATTTTAAAGAGCAAGAAAGGAAAGACCTTGGAAAACGCGGTTTGTGGGGATGTGGAAAAGTAAAGCAAATAGGTAATAAAGTTTGAGAGAAAATAAAAAAAAGGAAAAAAATATTGATGCAAAATTAGACCAAATAAAGACTGAGGCTATGCAGACAAGACTTGATGGAGACTGGTGAGAGAGGGACAGAACAGGATAGGAGAAGAAAAGAAAATTAAAGAAACAAATAAAACTGAGCTTTCTTTTTGACTTTGAATC |
| *MDH2* | Unigene15890 | GAGCGGTTGCATTTGGATAAGACCATTATTTAATTAGTTTATAGTGGCACGCATTCCAACTACAATAGCAACGTTTTCACGTCATCATTCATCCCAATTCCCAGCCTCCATTTCAATCCTAATTATTCAACCAAATTTAACCCAATTCCCAATTCCCAAATCACAATCAAAGAAAAATTAGACGAAAACGATGGATTCATCAGTAATCTCCGACGCCAATCGTCGCATCTCCAGAATCTCCTCCCATCTCCTCCCTCCCAGCAATCTCCAGATGGGGGAGGAAATTGATCGTTGCGGTCTGAGAAGAGAGATTTGCCGAGCGAAAGGCGCGGCGCCGGGATCCAAGGTCGCGATTTTGGGAGCCGCCGGCGGCATAGGCCAACCGCTCGCCTTGTTGATGAAGATGAACCCTCTCGTTTCGGTGCTTCACTTATACGATGTCGTTAATACCCCCGGCGTCACCGCCGACATCAGTCATATGGACACTGGTGCTGTGGTACGGGGATTCTTGGGCCAGGCCCAGCTGGAGGATGCCTTGACGGGTATGGACCTTGTGATCATCCCTGCGGGTGTTCCCCGGAAACCTGGAATGACTAGGGATGATCTGTTCAACATCAATGCTGGGATTGTGAAGACCCTTTGTGAAGCTATTGCCAAATGTTGCCCCAATGCTATTGTCAACTTGATCAGCAATCCTGTTAACTCCACTGTTCCCATTGCGGCCGAGGTTTTCAAGAAAGCTGGCACATTTGATCCGAAACGGCTTTTGGGAGTCACCATGCTTGATGTTGTTAGAGCTAATACTTTTGTGGCAGAAGTTTTGGGTCTTGATCCTAGGGATGTTGATGTTCCAGTAGTTGGAGGTCATGCTGGGGTTACTATTTTACCTCTTCTGTCTCAGGTTAAACCTGCATGCTCTTTTACTGATAAAGAAATTGACTACCTAACAGATCGCATTCAGAATGGTGGAACTGAGGTTGTTGAGGCAAAGGCTGGAGCTGGTTCTGCAACATTGTCTATGGCCTATGCTGCTGTCAAGTTTGCCGATGCATGCCTTCGAGGTTTGAGAGGAGATGCAGGGATAGTTGAATGTGCTTTTGTGGCTTCTCAGGTGACCGAACTTCCTTTCTTTGCATCTAAAGTACGGCTTGGACGTGGTGGCGTGGAAGAAATATACCCACTTGGTCCTCTGAATGAGTATGAGAGGGTTGGATTGGATAAGGCCAAGAAAGAGTTGGCAACAAGCATTCAAAAGGGAGTTTCCTTCATCAGGAAGTGAGTTCGTGCAGAGCAGCATTTTTCCTTCTTTGGAGATATTCTGTCTTATG |
| *PP2A2* | Unigene31643 | GGAAAAGAATGAGAAATGGATTTCTTCGCTCTTTTGCACACAAACAAAAGAATCCCAGGAAAGAGCAAAAAGAAGATTAAATTTTGGGGACCTTTCGGCAGTGTCATCACCTGGGCGTTGTCCATTTTCCTGGAAGAAAAAGAGAGGTAGGTGCACGTCTTTAAATTATTATTTTTATTATTAGTAGTACTTTTTATTTTTCCTTTTTATTTTTATTTTTTTGTGTTGTTGGGTTTTCATGATTTTTAAAAAGTAAAAATGGCTTCTTTCTTTTCTTCTCTCCTCTCTTTCAAAAAAGAAAAGGAAAAAATAAAATAATAATAAAAAAAAACATTATGGGATTTTTGAGGTTATCATGAGCTTCTTCTCTCTGGCCTCTCTCCTTCGCTAAAAGCTCTCTTCTTGTGAATTCACTCTTGCCCACAAGCTCAAAATCAGGAGATGATGATGAATCGAAGAATGAGATGTGTTCCTTAAAGACTACAAAGCAATATAGAACCGAAGACTTCTGTGCTGCTGCCGACCATCCTCCTTCCCCGGCAGTTTTCATTTGATATTTTGGCATATTAAACAAACTGCTATTTTTATTGATCCAGGAAGCAGCATTGCTACCATGCTGAAACAAATTCTAAGCAAACTTCCTCGTAAGTCTCCAAAATCAGATTCGCTAGATGGGGCAGGGAGTGATTCGGGCAGCAATTCCTCCAAGTTGGGTAATGGATTCCAATGTACAATTGGTGGGAGTTCTTTTTCTAGCAAGTTAAATGTTGTTAAACGAGTGTCTTCTGCGGTATTCCCAGCAAGCATCACAGCTGGTGCGGAGGTAGTGGAACCCCATCTATCCTTTAAAGATGTCTCAAATCCGCAGAAGCAGAATCTATTTATCAGTAAGCTCAACCTTTGTTGTGAGGTCGTTGATTACAATGATTCGGTTAAGCAAGATCTTAAACGTGACACATTGATAGAGCTCGTTGATTTTGTTTCTTCTGGATCTGCAAAGTTTACGGAATCAGCAATTTCAGCGATGTGTAAAATGTGTGCAGCCAACCTGTTTAGAGTTTTCCCACCTAAGTATCGCTCTAGCAGCACTGGCGGTGAAACAGAAGACGAGGAACCGGTGTTTGATCCTGCTTGGTCGCACCTACAAATTGTGTATGATCTGCTTCTTCGGTTTGTCAGCAGTACCTCACTTGACGCGAAGATGGCAAAGAAATATATTGACCATTCGTTTATTTTGAGATTACTTGACCTCTTTGACTCCGACGATCCAAGAGAAAGAGACTGTTTGAAAACGATTCTTCACAGGATTTATGGAAAATGCATGGTACATAGGCCTTTTATCCGAAAAGCCGTCAGCAATATCATCTATCGCTTTGTCTTTGAAACTGAACGGCACAATGGAATTGCGGAGCTGTTGGAGATTTTTGGCAGTGTAATTAGTGGTTTTGCAATGCCACTCAAGGAGGAGCACAAGATATTCTTGTGGAGGGCTCTGATTCCTTTGCACAAACCAAAATCAGTGGGAATTTATCATCAGCAGTTGACATATTGCGTCATACAATTCATAGACAAGGATCAAAAGTTGGCTAGTAATGTGATAAAAGGCCTGTTGAAGTACTGGCCAGTTACAAATAGCCAGAAAGAGTTGATGTTTTTAGGTGAGTTGGAAGAGATTTTGGAGATGACTAGTATGGTGGAGTTCCAAAAGATAATGGTTCCCTTATTTAGGAGAATAGCTTTCTGTCTCAGTAGCTCACATTATCAGGTGGCTGAACGAGCCCACTTAATGTGGAATAATGAGAGCGTCCTTAATCTCATCACGCAGAACCGCCAGGTGATTCTGCCGCTTGTCATCCCCGCCCTAGAGCGGAATACACAGAATCACTGGAACCAAGCAGTGCTAAACTTGACACTTAACGTGAAGAAGGTATTCTGCGAGATGGATGAGGAGTTAGTCCTTGCCTGTCAATGCAAGTTGGAGGGGG |
| *RPL4* | Unigene26991 | CACACTCTCACTTCAGCCCTAGCTCGTTTTTCCCTTTCGACTATATAAACCTCATTTCAGTTTCGGAAGAAAACCCTAGTTCATTCCCGAGAGAGAAAACAAAACCCTAGCCCCCCCACCCTTTCCCTTCCTCTCTCTCGTCTCCAATGGCAGCCACAGCAGCCGTCCGTCCCCTCGTCACCGTCCAGGCCCTCGAAGGCGACATGGCCACGGACGCCGCCCAAACCGTCGCCCTACCCGATGTCATGAAGGCCTCGATCCGTCCCGACATCGTCAATTTTGTGCACGCCAACATTTCAAAGAACAAGCGCCAGCCCTATGCCGTCAGCAGGCGCGCCGGTCACCAGACCTCCGCCGAGTCCTGGGGAACCGGTCGCGCAGTCTCCCGTATCCCCCGTGTTCCCGGCGGCGGGACCCACCGCGCTGGCCAGGGAGCCTTTGGAAACATGTGCCGCGGCGGAAGGATGTTCGCTCCGACCAAGATCTGGCGCCGCTGGCACCGCAAGATCAATGTGAACCAGAAGCGGTACGCCGTCGTTTCTGCCATCGCCGCCTCTGCTGTTCCTTCTCTCGTCCTGGCCCGCGGCCACAGGATCGAGTCAGTCGCGGAGTTGCCTCTCGTGATCAGTGACGCCGCCGAGGGAGTGGAGAAGACTTCTGCCGCGATCAAGGTTTTGAAGCAGATCGGAGCTTTTCCTGACGCCGAGAAGGCGAAGGACAGCCACGCGATTCGTCCTGGCAAGGGTAAGATGAGGAATCGCCGCTACATCAACCGCAAAGGTCCTCTGATTGTGTACGGAACCGAGGGAGCCAAGCTTGTGAAGGCTTTCAGGAACATTCCTGGAGTGGACATCATCAATGTCGAGAGGCTTAACCTTCTGAAGCTCGCTCCCGGTGGCCACCTCGGGAGGTTTGTGATTTGGACGAAGTCGGCCTTCGAGAAGCTTGACTCGATCTATGGATCGTTCGAGAAGCCTTCGGAGAAGAAGAAGGCTTACGTGCTTCCCAGGCCGAAGATGTTGAATGCGGACTTGGCGAGGATCATCAACTCCGATGAGGTTCAGTCTGTCGTGAGGCCGATCAAGAAGGAGGTAAAGAGGGCACCACTGAAGAAGAACCCTCTGAAGAACCTCAATGCCATGTTGAAGCTCAACCCCTACGCCAAGACCGCCAAGAGGATGTCTTTATTGGCTGAGGCCCAGCGCATCAAGGCCAAGAAGGAGAAGCTTGACAAAAAGAGGAAGCCCGCCGCTTCTAAGGAGGAGGCTGCTGCTATCAGGGCTGCAGGAAAATCGTGGTACCAGACTATGATCTCAGACAGTGACTACACCGAGTTTGAAAACTTCACCAAGTGGTTGGGAGTATCTCAGTGATTTTCTGCTTTTTCATATCTCGAGACATTAGTTTGATACTTTTGTTTTGGTGTTAATTTTGTGTCCTTTTTCTGCTTTGAAGCGGGAGGAAGATTAGTGGAATAATAGGGGAGAATTTTGACTGGATGTCTTTCTTCCTTTTCTTGTTCTCTGTTTTCCTTCCAGAGATTGAAGAGTTTTTATTATCAGTACTGGATTCAAAGATTTTAGATTACATAGTTTAGTTGGTCAAATTCAGTTTCTCTTGGCAAATCTAAGCTGGTATGAGATATTGACATTCTATTTATGTTATTACATGAAACAATTTTCCAATTGGCGTGGCTTGTTTTGGTGTTTTTGCCCATGATTAGGTTTCCTTATCAACACACTCAGTTGATTGTTAGTTTTATGAAAACCTTTTTGTAGTTCTTCCCGGCATATTCTAGCTTATATGGCAAAGAACTTTTTGGACCATTTAACCAAAAAATGATCAGACTACAGACTGCTGTCTTGATGCTAAGATAACTTCTCAACGTATTGTTTTAAGTTGACATTGTTTCTCATGCGTGTTTTTG |
| *RPL35* | CL8011.Contig2 | ATCGGAAGAAATACAATGGCGAGAATCAAGGTTCACGAGCTGAGGCAGAAGACGAAGGCGGAGCTGCTGAACCAGCTCAAGGATCTCAAGGCGGAGCTCGCTCTCCTCCGCGTCGCGAAGGTCACCGGCGGTGCCCCAAACAAGCTCTCCAAGATAAAGGTGGTTAGGCTTTCGATCGCGCAGGTGCTGACGGTGATTTCACAGAAGCAGAAGTCGGCGCTTAGAGAAGCTTACAAGAACAAGAAATACTTGCCTCTCGATCTCCGTCCCAAGAAGACCAGGGCCATCCGCAGAAGGCTTACCAAGCACCAGGAATCTTTGAAGACTGAACGTGAGAAGAAGAGAGAGTTGTATTTCCCCAAGAGGAAGTATGCCATTAAGGTGTAGGGTGGGTTCCATTTCATTAGGTCATTATGGTTTTTATTGTTTTACAGACCTATTTGGTTGAGGCAAGTTTTGACGTTTTTTTTTCCATCACTCTTTGATAGTTATGTTACTGGACCTGATGTTGTGTCGTTCAATTTCATTTCTGTAACTTGGGAAGACATGTCTCAACTAAACCAAGTTATATTATTTTCAAGAATGATAGTTTAAGATTGATGTTCATGTTTGATTAATGCTCCCCTTGTTTCTCGCTATCGCTAGTTAGCTGTTTAGTTCTAACACTTGATTTTGGTTTTTCAATCCTTTTGATGGGAACAACTGCAAGACATGTTTGAAGCATAATTAGGATTTTATTTGGCTTG |
