# Supplementary material for: Identification and validation of reference genes for qRT-PCR analysis in mulberry (Morus alba L.)
Source: PLoS One. 2018 Mar 15;13(3):e0194129. doi: 10.1371/journal.pone.0194129 (PMC5854264; doi:10.1371/journal.pone.0194129)
Supplement: S2 Table — (DOCX) [file pone.0194129.s004.docx]

S2 Table. RPKM (reads per kilobase of exon per million reads mapped) of the 18 candidate reference genes selected from our transcriptome database in three mulberry varieties after infection with *R. solanacearum* for various time points^1^.

| **Gene Name** | **Gene ID** | **Mulberry Varieties** | | | | | | | | | | | |
| --- | --- | --- | --- | --- | --- | --- | --- | --- | --- | --- | --- | --- | --- |
|  |  | **YS283** | | | | **YSD10** | | | | **KQ10** | | | |
|  |  | **RPKM-0 d** | **RPKM-1 d** | **RPKM-3 d** | **RPKM-8 d** | **RPKM-0 d** | **RPKM-1 d** | **RPKM-3 d** | **RPKM-8 d** | **RPKM-0 d** | **RPKM-1 d** | **RPKM-3 d** | **RPKM-8 d** |
| *ACTIN4* | Unigene23275 | 322 | 320 | 344 | 303 | 403 | 460 | 313 | 224 | 296 | 337 | 321 | 321 |
| *TUB2* | CL1595.Contig6 | 273 | 236 | 266 | 257 | 316 | 234 | 301 | 228 | 282 | 212 | 233 | 262 |
| *TUB3* | CL8311.Contig1 | 131 | 149 | 187 | 167 | 186 | 117 | 158 | 12 | 213 | 108 | 158 | 219 |
| *TUB4* | CL2672.Contig2 | 97 | 120 | 115 | 118 | 149 | 159 | 141 | 113 | 113 | 156 | 133 | 115 |
| *UBI3* | Unigene18627 | 701 | 381 | 376 | 383 | 429 | 343 | 346 | 281 | 401 | 356 | 334 | 307 |
| *UBI4* | Unigene5350 | 256 | 289 | 277 | 284 | 297 | 328 | 333 | 355 | 304 | 360 | 318 | 263 |
| *UBI5* | CL1942.Contig2 | 194 | 225 | 217 | 185 | 202 | 213 | 193 | 153 | 197 | 219 | 223 | 214 |
| *EF1α1* | CL4198.Contig2 | 5611 | 7094 | 5647 | 5279 | 5616 | 1318 | 6866 | 3537 | 4874 | 7370 | 6186 | 5789 |
| *EF1α3* | CL272.Contig1 | 1687 | 1435 | 1121 | 936 | 1235 | 216 | 1337 | 684 | 1209 | 1338 | 1180 | 1082 |
| *EF1α4* | CL155.Contig3 | 1257 | 1062 | 901 | 806 | 686 | 67 | 685 | 354 | 804 | 729 | 862 | 1111 |
| *GAPDH1* | CL3772.Contig5 | 1962 | 1500 | 1528 | 1464 | 1050 | 82 | 1149 | 225 | 1325 | 1359 | 1745 | 2015 |
| *GAPDH2* | CL131.Contig2 | 1272 | 1227 | 1051 | 1019 | 775 | 67 | 949 | 524 | 1060 | 1077 | 1072 | 1235 |
| *CYP1* | Unigene15318 | 8601 | 8421 | 9119 | 7710 | 3964 | 581 | 7473 | 2823 | 4540 | 6282 | 7882 | 9373 |
| *MDH1* | Unigene16243 | 85 | 134 | 104 | 96 | 85 | 133 | 117 | 50 | 81 | 112 | 116 | 90 |
| *MDH2* | Unigene15890 | 45 | 33 | 43 | 41 | 40 | 35 | 41 | 54 | 45 | 37 | 38 | 40 |
| *PP2A2* | Unigene31643 | 43 | 33 | 59 | 59 | 59 | 30 | 39 | 16 | 60 | 38 | 51 | 57 |
| *RPL4* | Unigene26991 | 170 | 308 | 224 | 161 | 200 | 298 | 262 | 143 | 156 | 270 | 215 | 226 |
| *RPL35* | CL8011.Contig2 | 379 | 393 | 306 | 236 | 264 | 448 | 325 | 227 | 294 | 377 | 298 | 285 |

^1^Plants were infected with *R. solanacearum* for the indicated number of days before tissue was harvested and sampled.
