# Supplementary material for: Identification and validation of reference genes for qRT-PCR analysis in mulberry (Morus alba L.)
Source: PLoS One. 2018 Mar 15;13(3):e0194129. doi: 10.1371/journal.pone.0194129 (PMC5854264; doi:10.1371/journal.pone.0194129)
Supplement: S3 Table — (DOCX) [file pone.0194129.s005.docx]

S3 Table. Information of *MaCHSs, MaERF, MaDELLA, and MaJAZ* genes.

| **Gene name** | **Gene description** | **Accession number/RNA-Seq**  **number** | **Primer sequence (5′–3′)** | **Amplicon length (bp)** | **Gene sequence (5′–3′)** |
| --- | --- | --- | --- | --- | --- |
| *MaCHS5* | Chalcone synthase 2 | CL3598.Contig1 | F: TGACGGGCACTTGAGGGAAATAG  R: TATGGAGTTCCAGTCGCTGATCC | 133 | GCTAGCTAATTAATTCAGATTGCAAATTAATAGCTAAAGCACCAAGCTACCTATAGTAATTTGTCATAATCTCTAAATTAATTAGCTAGCAAAACCCTTATCCCAGCTAGCAAAGTCAACCGTCATTATATTTAATAATTAATATTAGCTAGCATGGCGACACCCTCCTCCGTCCAGGAAATCCGAAAGGCTCAACGGGCCGATGGACCGGCCGCCGTGCTGGCCATCGGCACCGCAAACCCGCCCAACTACTTCTTCCAGACGGATTATCCTGACTTCTACTTCCACATCACCAACAGCGACCACAAGACTGAACTCAAGGACAAGTTTACACGCATGTGTGAGAAAGCAATGATAAGGAAACGTCACATGTACTTAACAGAAGAAATTCTTAAGGAAAACCCAAAGATGTGCGAGTTCATGGCGCCGTCACTGGACGCCCGGCAAGACATAGTGGTGGTTGAGATACCGAAGCTTGGCAAAGAGGCGGCTGTCAAAGCCTTAAAGGAGTGGGGCCAGCCAAAGTCAAAGATCACTCATCTCATCGTCTGCACAACCTCCGGCGTCGACATGCCCGGCGCTGACTACCAGATAACCAAGCTCCTCGGCCTCCGCCCCTCCGTCAAGCGCTTCATGTTGTACCAGCAGGGCTGCTTCGCCGGCGGCGCCGTCCTCCGCCTTGCCAAGGACTTGGCCGAGAACAACAAGGGTGCCCGCGTCCTCGTCGTCTGCTCCGAGATCACAGCCGTCACCTTCCGCGGGCCCTCCGAGCTCCACCTGGACTCTATGGTTGGCCAGGCCCTCTTCGGTGACGGTGCCGCCGCCGTCATTGTTGGAGCCGAACCGGACTTGTCGATCGAAAGGCCGATCTTTCAGCTAGTGTCGGCAGCGCAGACGATACTTCCGGACTCGGAGGGGGCAATTGACGGGCACTTGAGGGAAATAGGGCTTACGTTTCATCTGCTGAAGGACGTGCCTCTGTTGATATCAAAGAACATAGAGAAGAGTCTGGAGGAAGCATTCACTCCGATCGGGATCAGCGACTGGAACTCCATATTTTGGATAGCGCATCCGGGAGGTCCGGCGATTCTTGACCAGGTGGAAGCCAAGGTAGGGCTGCACAAGGAGAAGCTGAGGGCCACTCGCGAGATGTTGCGCGAGTACGGAAACATGTCCAGCGCCTCTGTACTCTTCATATTGGATGAGATGAGGAAGAAGTCAGCGGAGGAAGGGAAGTCAACCACCGGAGAAGGCCTCGATTGGGGAGTCCTCTTTGGCTTCGGCCCGGGCTTGACCGTAGAGACGGTCGTGCTCCACAGCCTTCCCATCAATAATTAATTAATCGATCCATGGTATAATTACATGATCTATGACAGATCAGAAAGATAAAGAAAGCTACATTTTTTTTTTTTTAATTTTCCTTTTTAATGTTATACGTGTTTCAAAAATAAGTACACCCCACGGCGCGAATGATAATAGATCCAAGACAGATCTACGTGCATTGTTAGAAGGTAATTAATAATATTTTGATGAAGGCGCTTGTAATAATTTAAGTTGTTATATTGGAATAATTTGCAATATTCAAAATATTCACATCATGAGATGGCGCTGGCGTTTCTTAAGTTACAACATATATCAATTAATCTCAACGTCACATATGTAGGATTATATGGTATATACGCAGGCAATGTTATTTTGTAACTCAGAAAGAGCAATTATTGTTGAACC |
| *MaCHS6* | Chalcone synthase | Unigene23165 | F: CACCAACAGCGAGCACCTGATTC  R: TTCATAAAGCTGCAAAGACTAGGGT | 135 | CATTAACAGTGTGGATTTTCAAAACTCAAAAGTTATAACTCAAACTTTTTATGTGAGAGAAAATAACAAAAAAATTGAATTTAAATTTTTGTGAGAGGAAACACTTCGTGATTTGAGTTGTGAAAATAACAAGGCCTAACTGTATCTACGGATTTGTATGTGTGATGGAATCGGTCAAAGAATTCCGAAAGGGTCAGCACTCTGAAGGTCCGGCTTCCATCCTGGCAATTGGTACTGCCAATCCATCCAATTGTGTTTCGCAAGCTGATTATCCTGATTTTTTGTTTCGCACCACCAACAGCGAGCACCTGATTCAGCTAAAAGAAAAATTCAAACTCATGTGTGAAAAAACAATGATAAGGAAACGACACATGTACCTGACTGAAGAAATACTCAAGAAAAACCCTAGTCTTTGCAGCTTTATGAAACCATCTCT |
| *MaCHS7* | Chalcone synthase | CL4405.Contig1 | F: GACGGATTGGAGTGGGGAGTTCTTC  R: CCAAGCATTTTTATAGAATTTACGCA | 109 | GACGGATTGGAGTGGGGAGTTCTTCTTGGGTTTGGGCCGGGACTCACTGTGGAGACTGTGGTCTTACAGAGTGTCCCTATTATTGCGTAAATTCTATAAAAATGCTTGGGGTACCACCAATCTTAAGTGGCATAACTAATGAAAGGTGGATCCTTGTTCTATATTCTAAGATGCGGGACACCTTGTATTAGTGTATGATGCCTAAATGTGTCTTCTACATTATTCCAGATTATTTCACACAAAG |
| *MaERF* | Ethylene response factor 6b | Unigene5487 | F: AACGACCCTACCTCCCAACTTCC  R: GCCTCTTCCTCACTCCTCTGTAA | 247 | AAAACGACCCTACCTCCCAACTTCCACCTCTTATTCCCTCTCTTTTCTCTGCCCAAAGCAAACCAAACACCGTTTTTTCCCTTTTCTCCTTTCATTTTCATTTTTTATACAAATATAAAACAAGCTCTGGGGATTTTATAATTGAAATGGCGCCGAGAGAGAAGATGGCCACCGCCGCCGCCACCACCGCCGCCGTTAAAGTTAACGGCAACGCGAAGGAGGCGCATTACAGAGGAGTGAGGAAGAGGCCGTGGGGAAGATACGCCGCCGAGATCAGGGACCCCGGCAAGAAGAGCCGCGTCTGGCTCGGCACCTTCGACACCGCT |
| *MaDELLA* | DELLA protein | Unigene15719 | F: ACCTTCAAGCCTTACTTCAACAGAA  R: AAACAAGCTAACAACACGGACACA | 149 | CCCGGCTTTCCATTTCTCTCGTCAAAACCCTTTCTTCCACCAACTATTTTCACTTCTCAAAGAGTCTCAAAATCTCAGATACAAAATCACTCTTTTTCTCTCTACATCTCCTTGTTCTTGGAATGAATTACAAACCCAAAACAAACCCATCTGATTTCCGATAATCAGAAACCAGAGAGAGGGGGGAAAAAGGGAATCCCATTTTATTTCCACTATGGGGCCGTACGACTCCCCCAACTCGGCCGGCAGCACCAGCGGCAGCTCATCCTCCTCGTTGCCGAAGCCCCCAAACGAAATCGACGGCCTCCTCGCCGGCGCCGGCTACAAGGTCCGCTCCTCCGATCTACGACATGTCGCTCAACGCCTCGAACGCCTCGAGACTGTCATGTCCGTCAACTCCCCAACCGTTGACATCTCCCAACTCGCCTCCGACGCCGTCCACTACAATCCTTCCGATATCGGCTCTTGGGTCGACTCTCTCCTCTCCGAATTCTCTTTCCCC |
| *MaJAZ* | Protein JAZ2 | Unigene31104 | F: TTCCACCAACTATTTTCACTTCTCA  R: CCCTCTCTCTGGTTTCTGATTATCG | 207 | TCTCTCTTTGCTAATCAGTAGATATGTCGGAAAATGAGAGGTTTGTGAACAGGAAGGCCGGCAAGGCGCCGGAGAAGTCGAATTTCGCTCAGACCTGTACTCTTCTGGGCCAGTTCTTGAAGGAGAAGAGGAATCTTGGCGTCGTAATTGGGAATGCCGAAATTGCAAAAGAGAAATTACCGGAGACGTCGTCGTTCCGACCTCCGATGGCGACGAAGAATTTCTTTACCAACATGGGGAACCAAGCTCAAGCTGCGCCCTCTGCAGTACAAAACGGTACCGTTTCAGCGTCGACAATGGTGAAATCCTCCGATTTCTTTCAACAGTTCAAGGGTTTTGGTACTCCTCCTTCTTCTGGTCCCAATGTTGAAGATGCTAATAAGCCTGATCTAAGGAAATCGGCAAGTACTGCTGTTGTTAAGCCTGAAACTGCACAGATGACGATATTCTACGCCGGCCAAGTTCTCGTGTTCAACGATCTCCCGGCCGACAAAGCCAACGAAGTCATGGCTTTGGCCAGAAAAGGAAGCTCAAACATTAGCGGTTCTGTTTCTACTTCCACTCCGGCAGTTGTGGAGAAGATTGATTTGGCCAAGCCTGCTGCTCCTGAAAGCAACGTTACCCTAATTACCAAGTCTGATAATAGCAATAACAACATTAATATCAATGATT |
