# Supplementary material for: Identification and validation of reference genes for qRT-PCR analysis in mulberry (Morus alba L.)
Source: PLoS One. 2018 Mar 15;13(3):e0194129. doi: 10.1371/journal.pone.0194129 (PMC5854264; doi:10.1371/journal.pone.0194129)
Supplement: S1 File — (PPTX) [file pone.0194129.s006.pptx]

## Slide 1
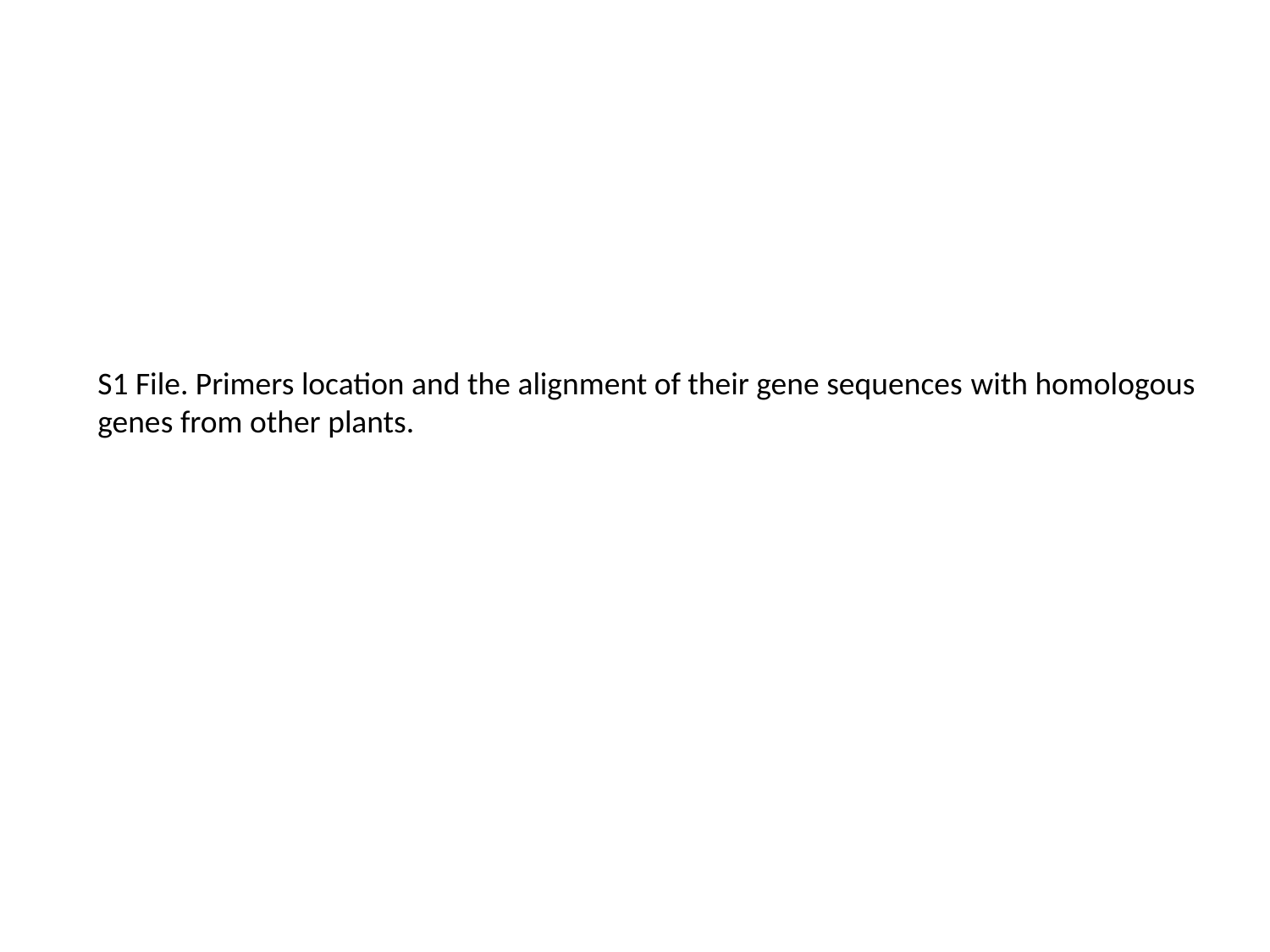

S1 File. Primers location and the alignment of their gene sequences with homologous genes from other plants.

## Slide 2
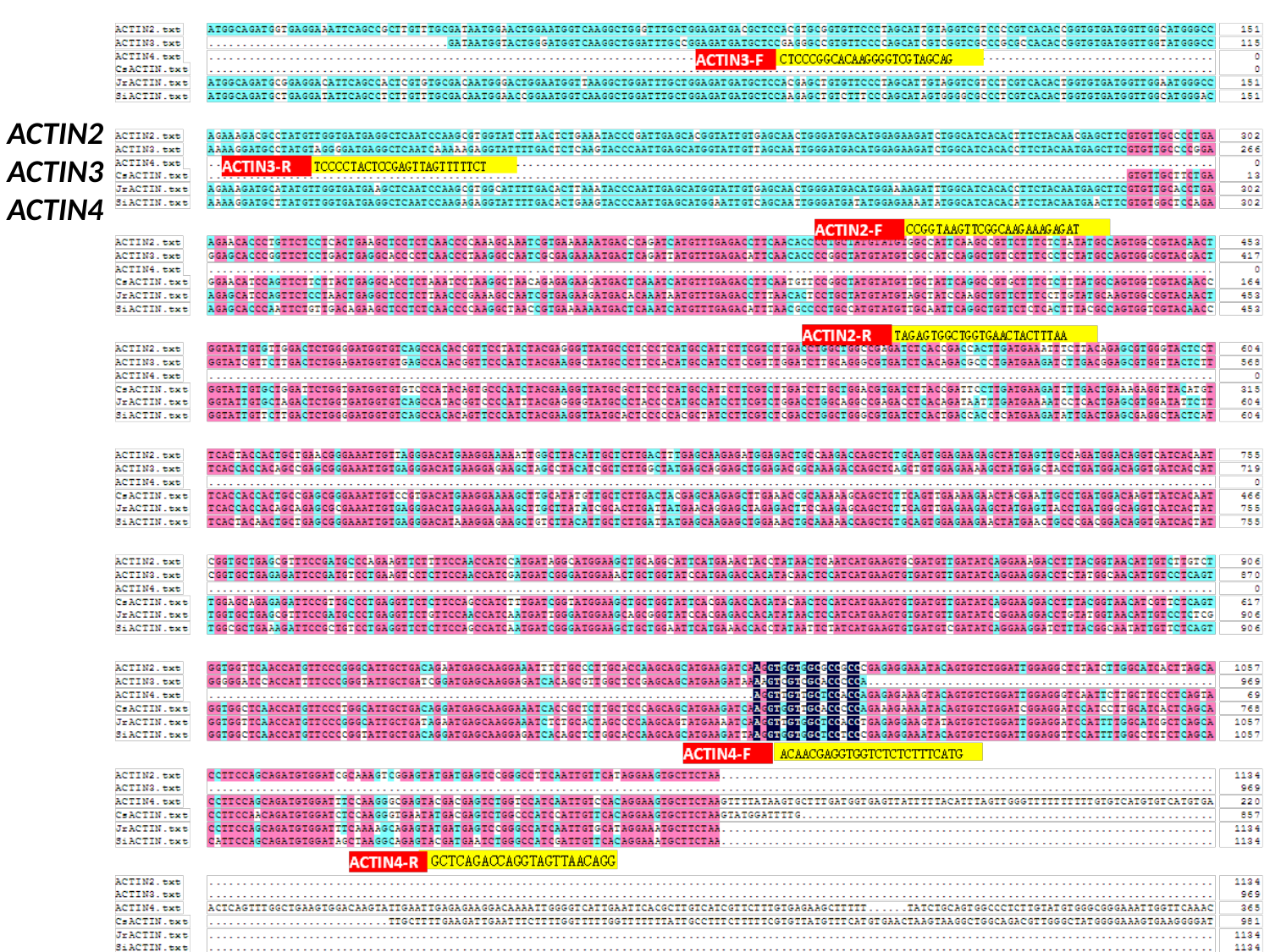

ACTIN2
ACTIN3
ACTIN4

## Slide 3
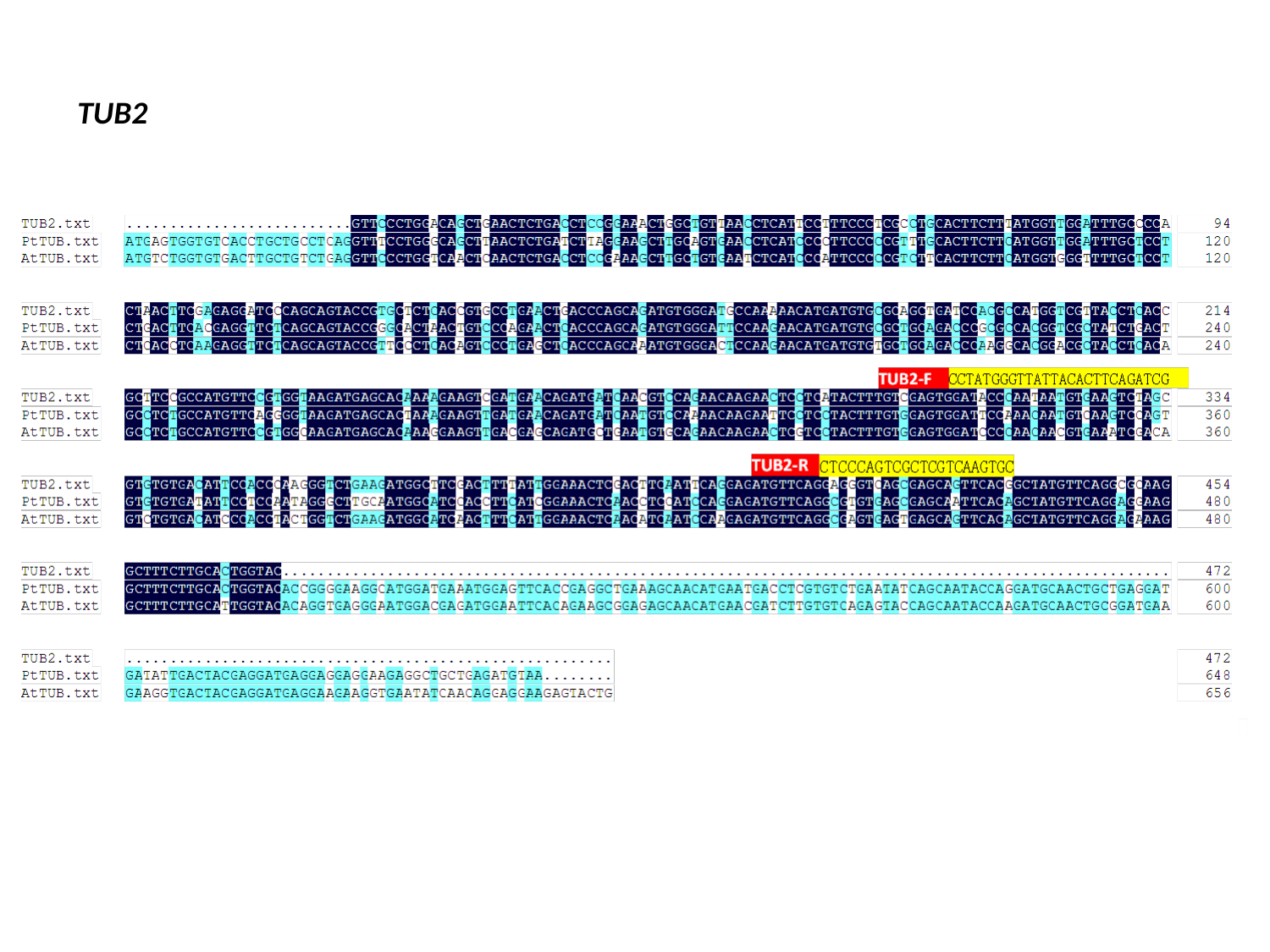

TUB2

## Slide 4
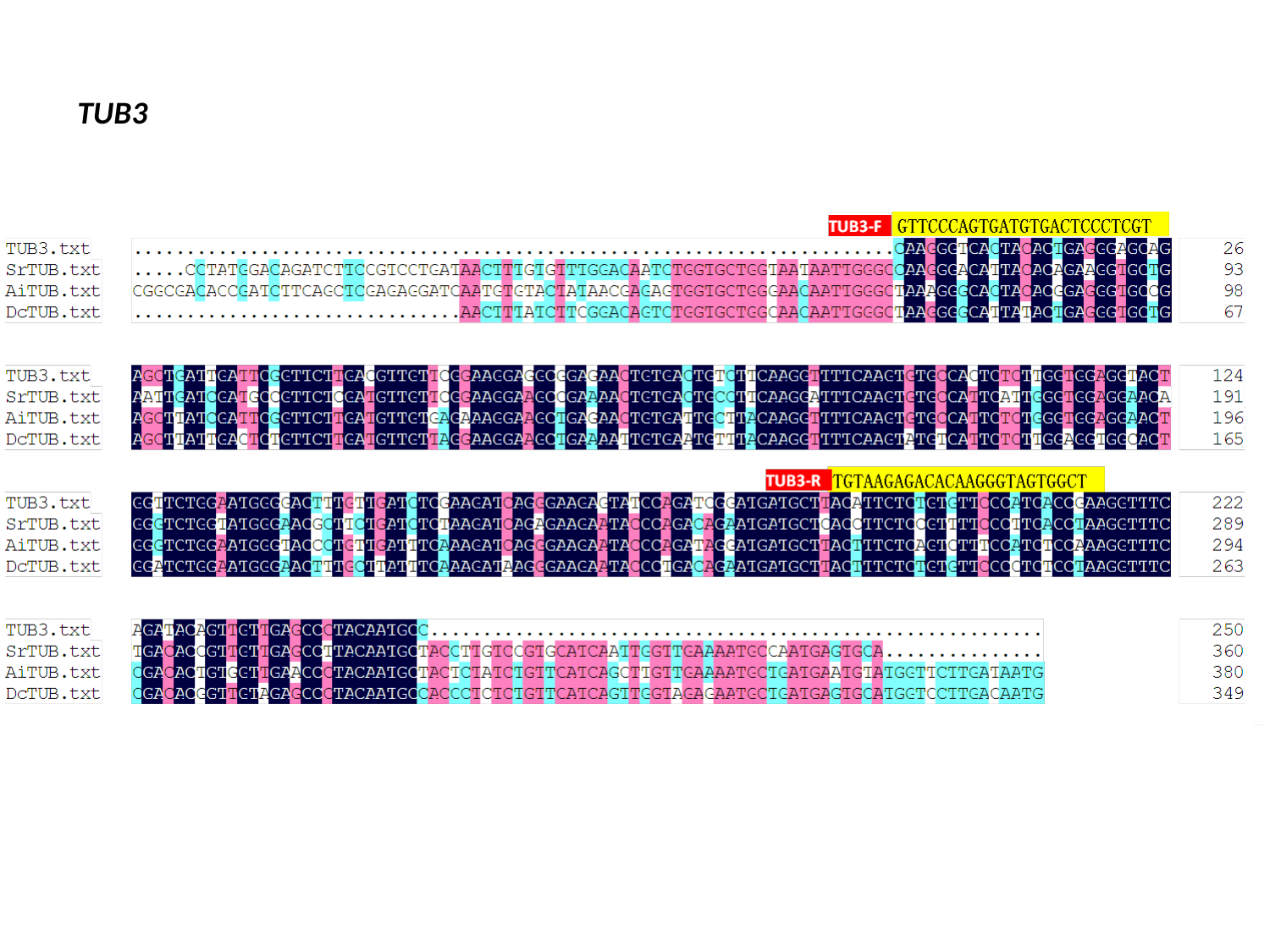

TUB3

## Slide 5
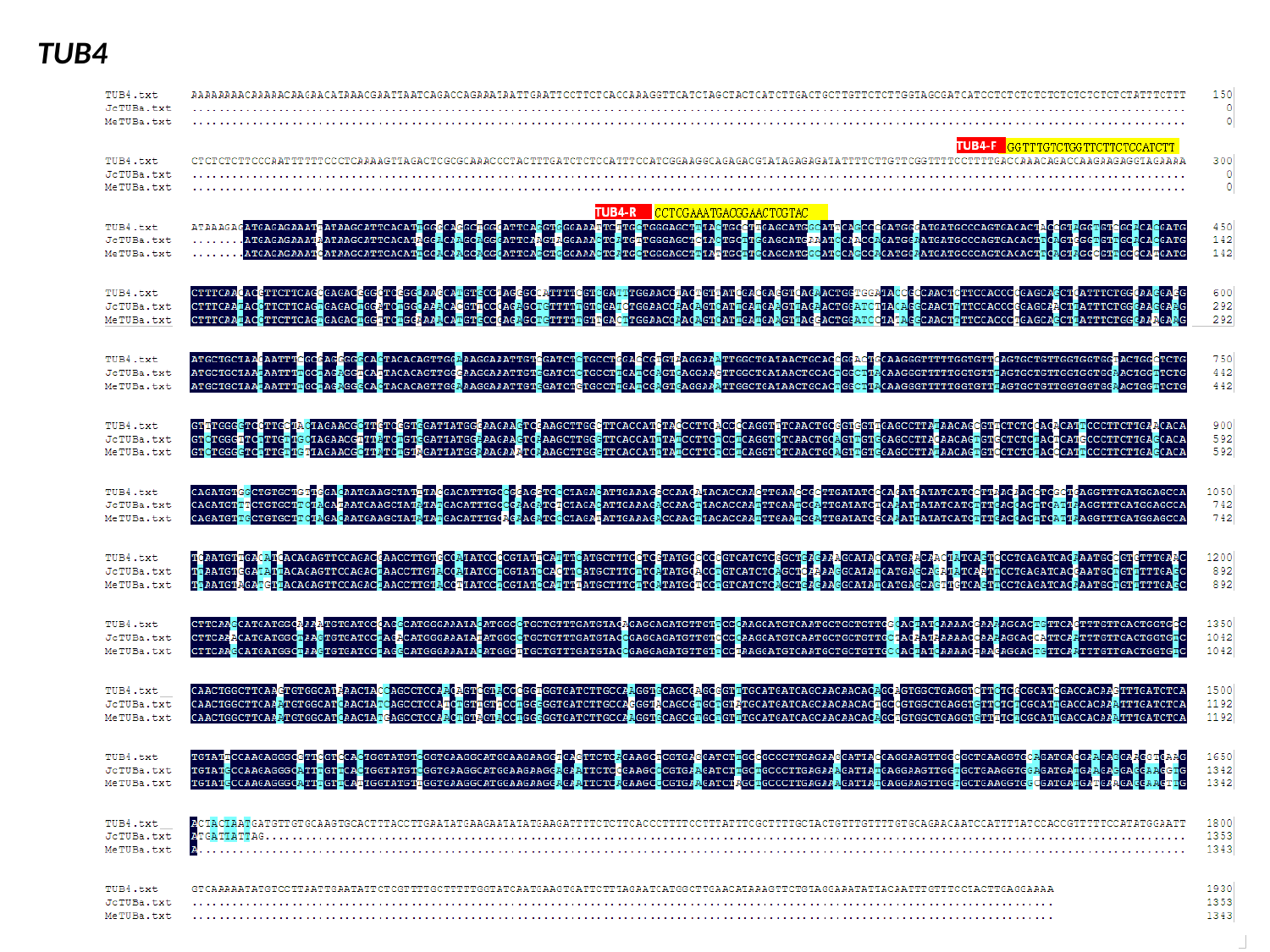

TUB4

## Slide 6
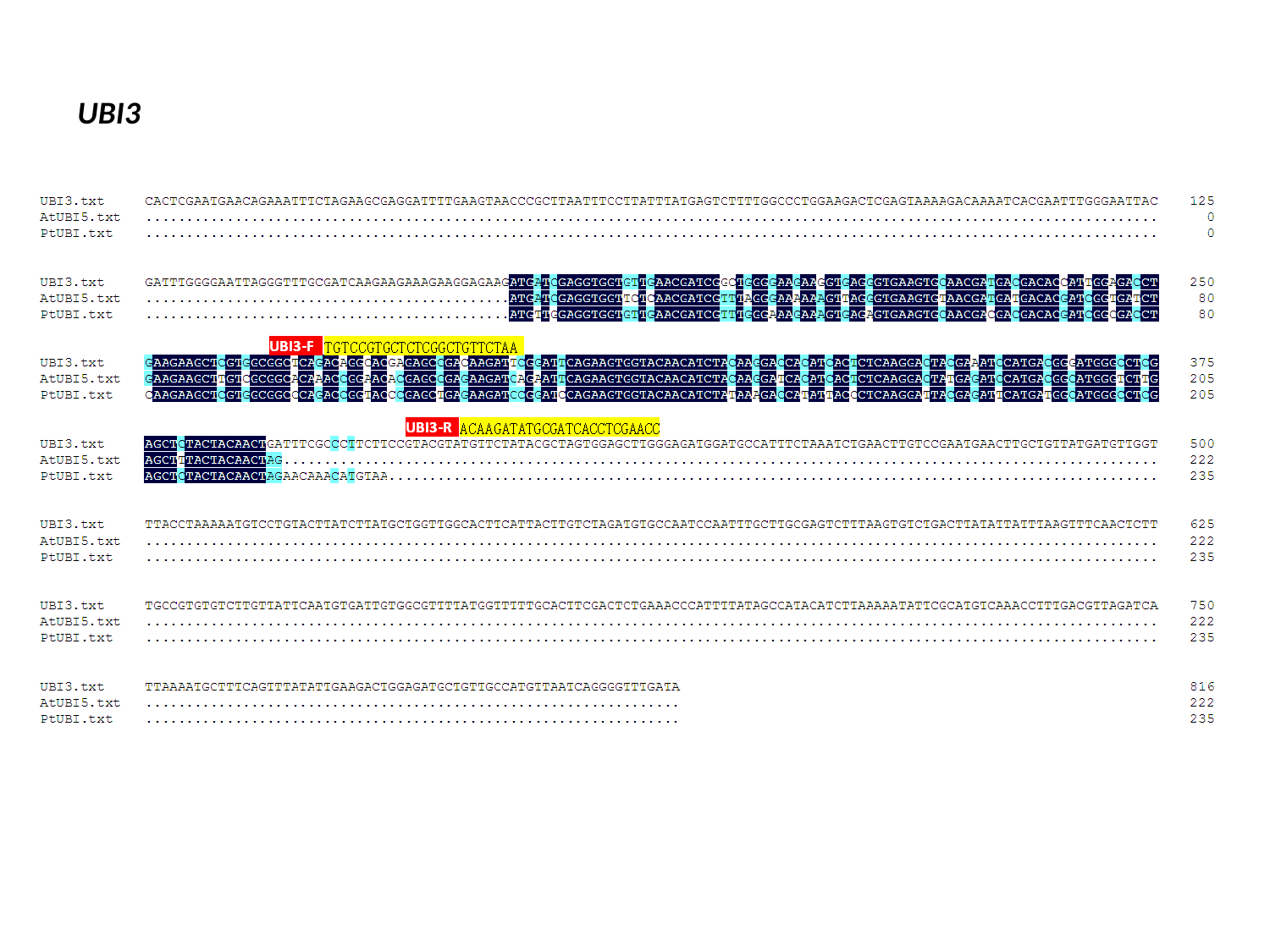

UBI3

## Slide 7
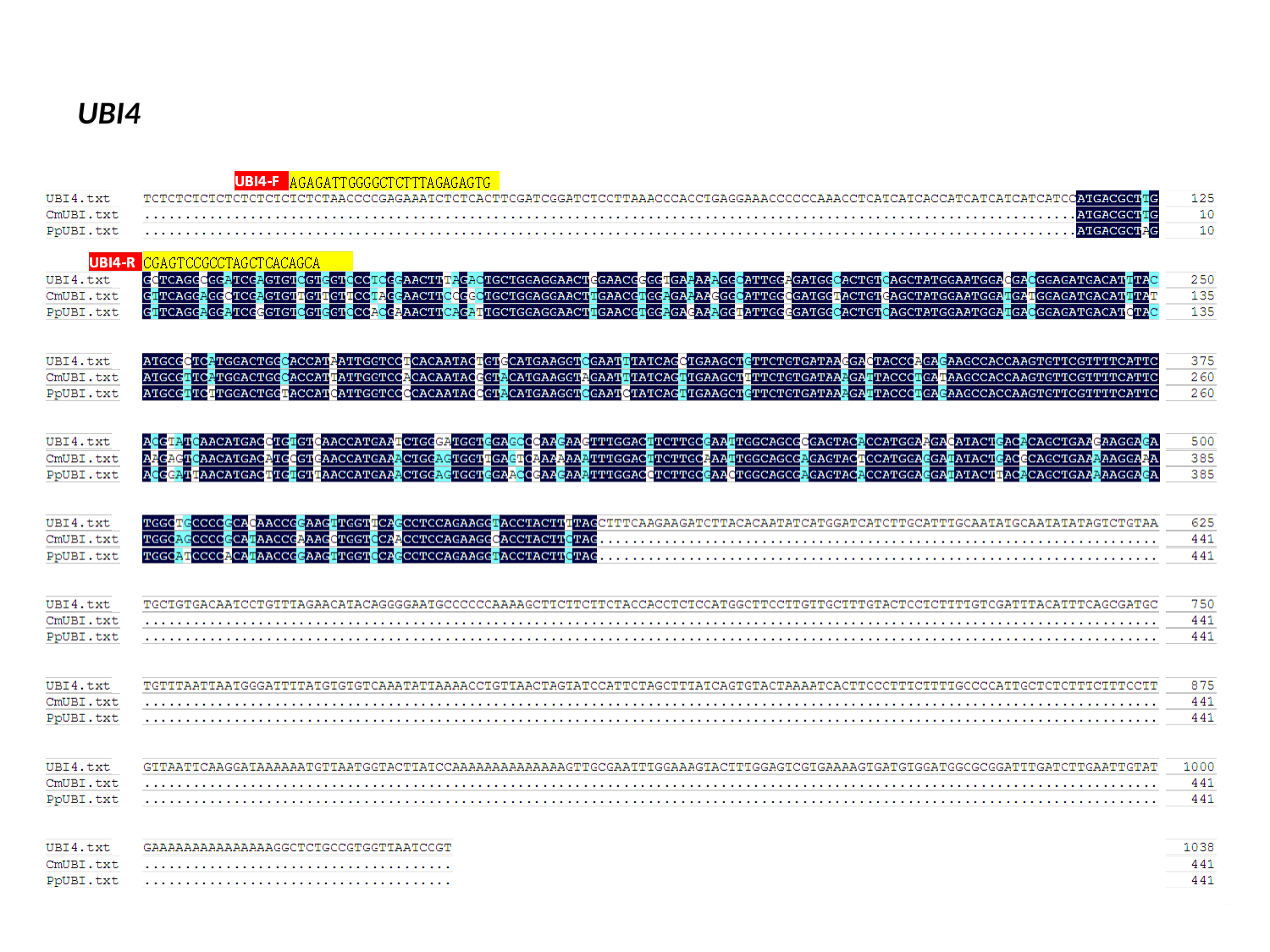

UBI4

## Slide 8
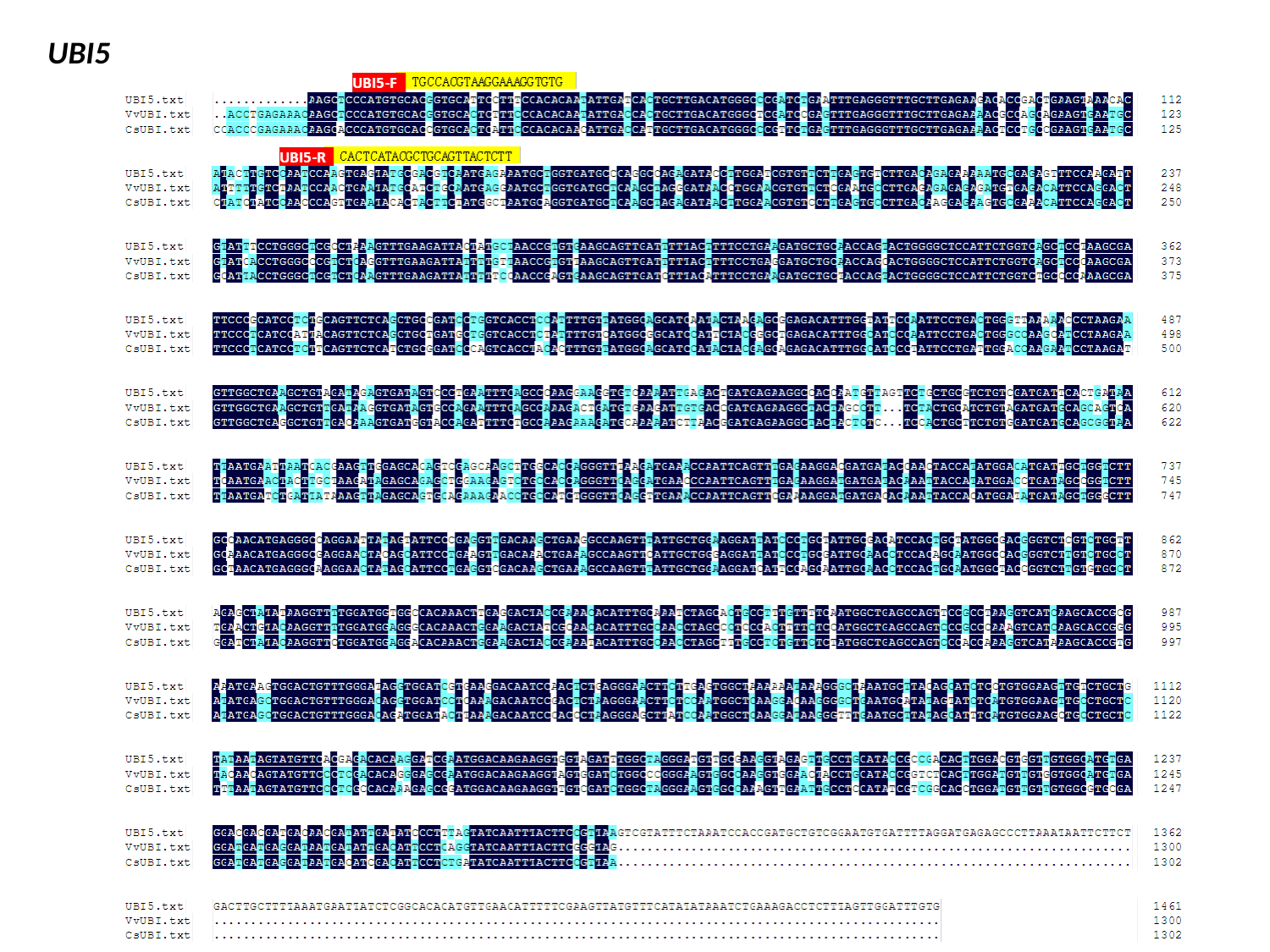

UBI5

## Slide 9
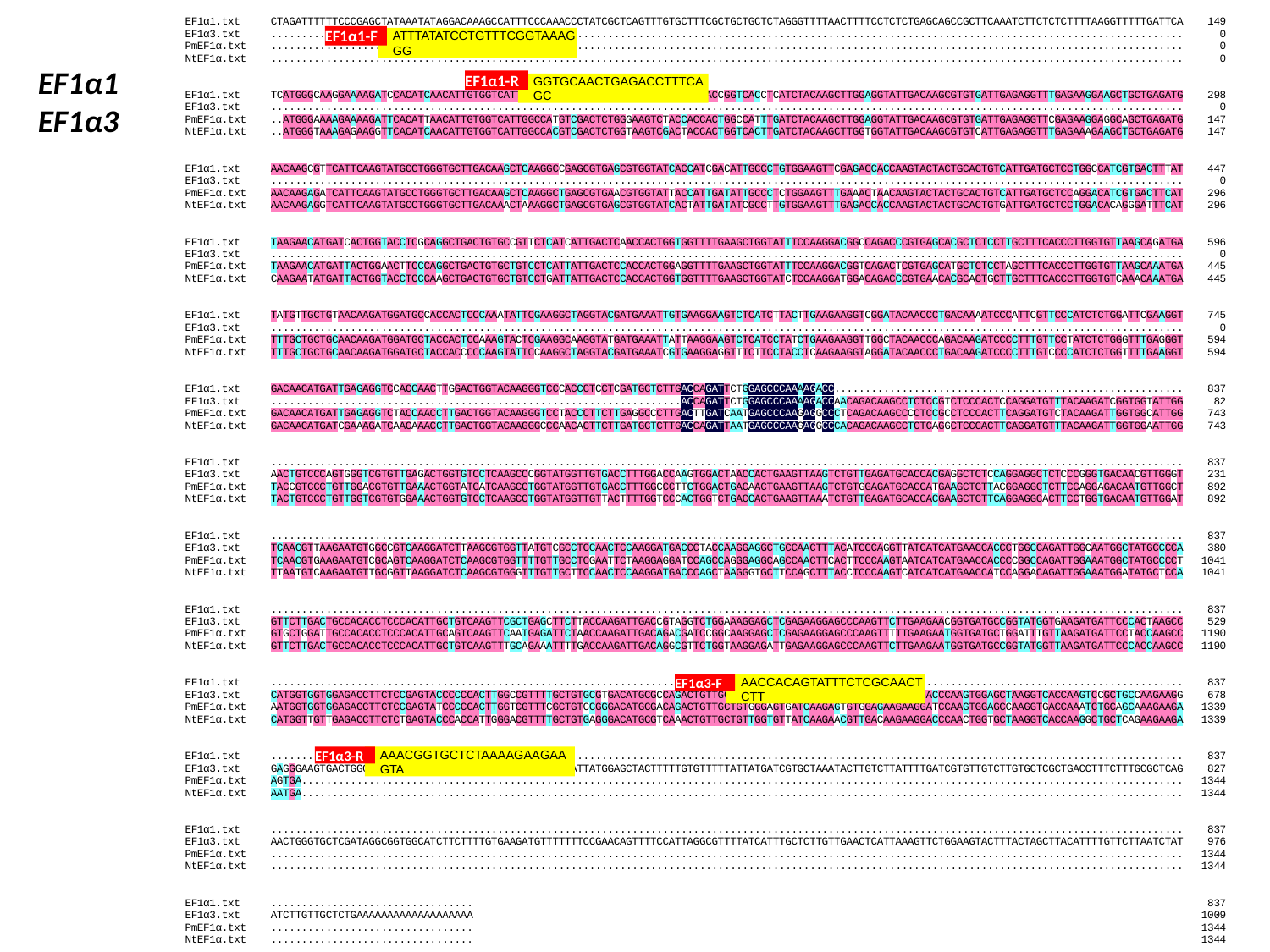

EF1α1-F
ATTTATATCCTGTTTCGGTAAAGGG
EF1α1-R
GGTGCAACTGAGACCTTTCAGC
EF1α3-F
AACCACAGTATTTCTCGCAACTCTT
EF1α3-R
AAACGGTGCTCTAAAAGAAGAAGTA
EF1α1
EF1α3

## Slide 10
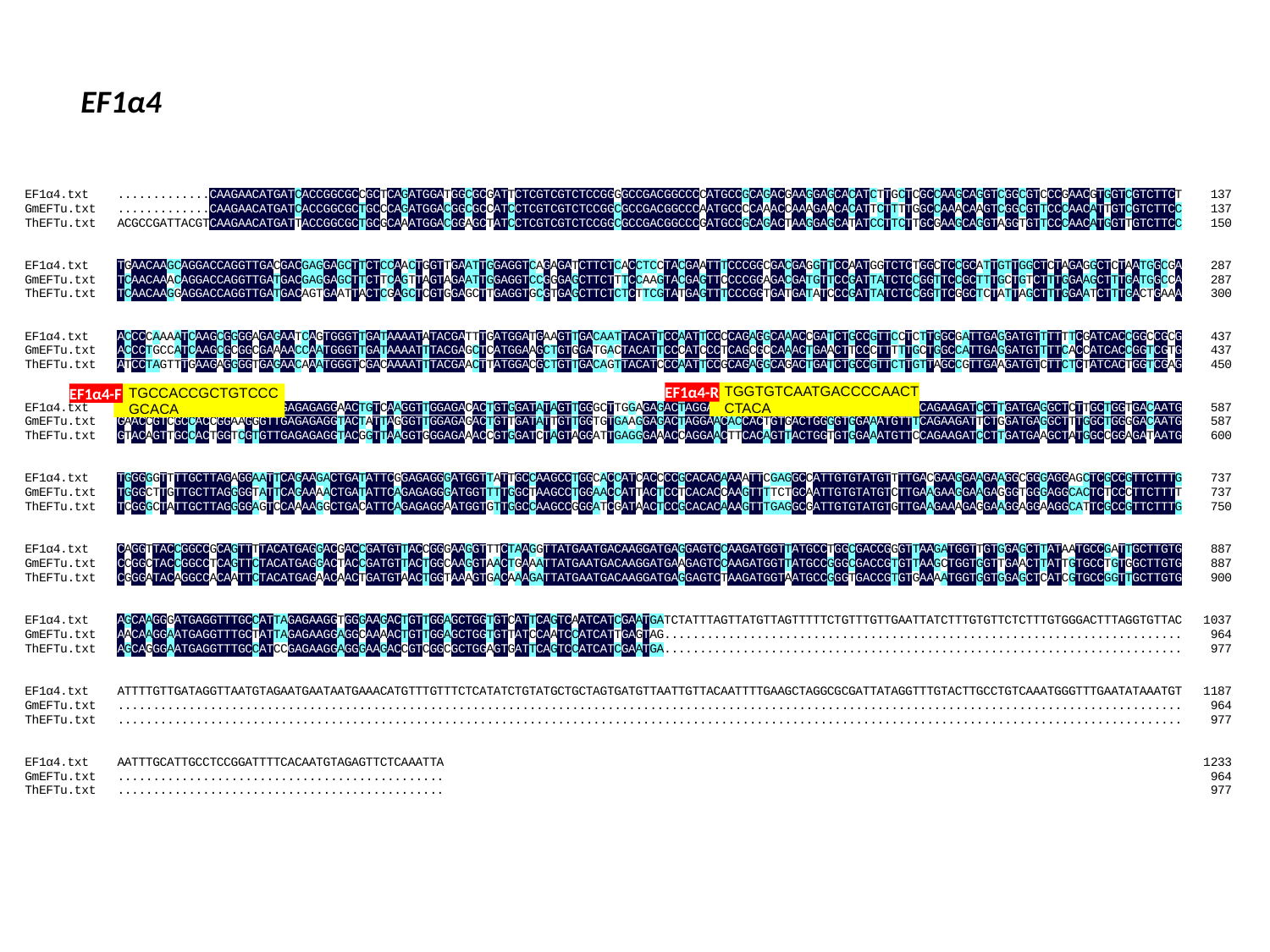

EF1α4
EF1α4-R
TGGTGTCAATGACCCCAACTCTACA
EF1α4-F
TGCCACCGCTGTCCCGCACA

## Slide 11
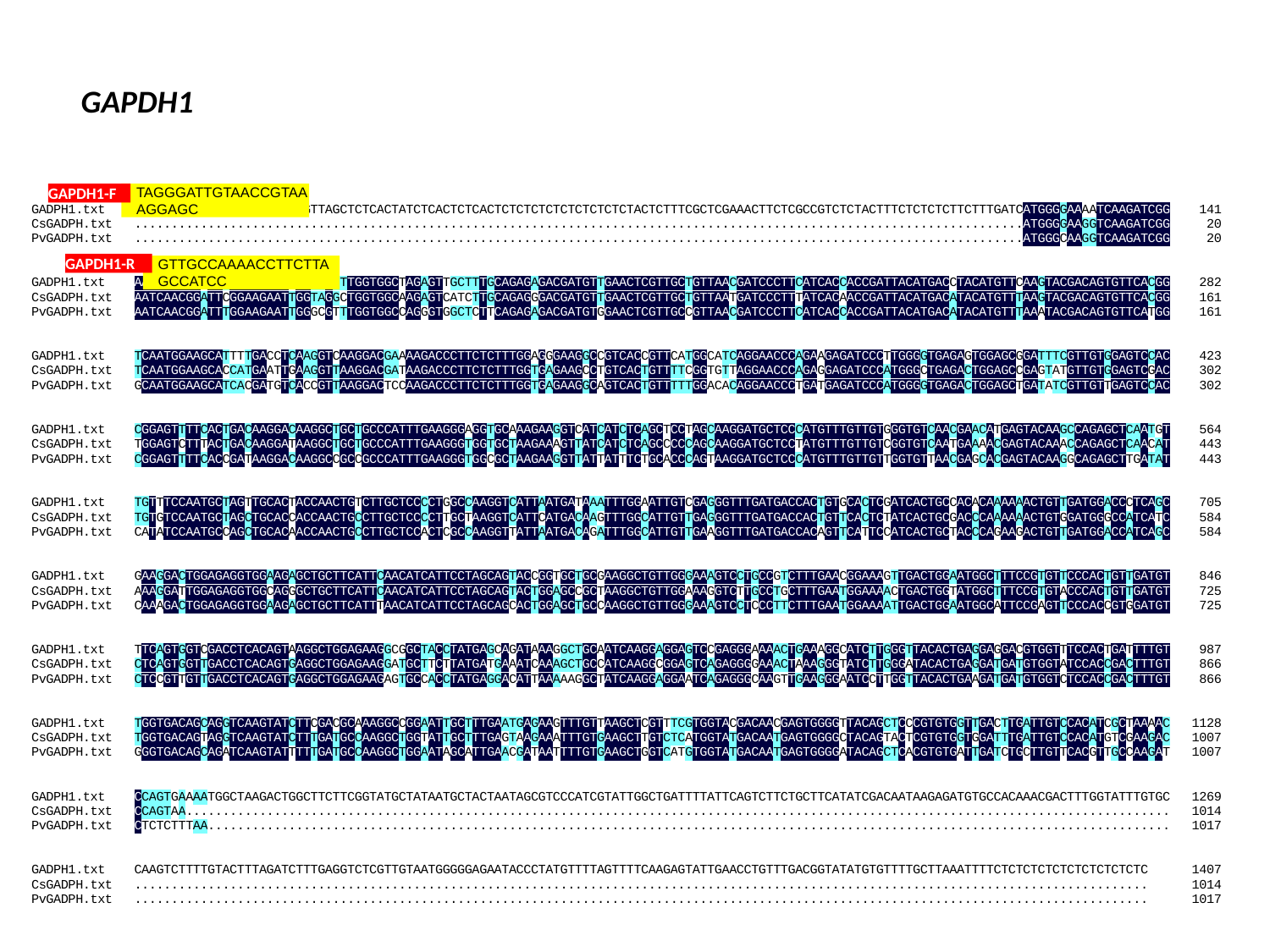

GAPDH1
GAPDH1-F
TAGGGATTGTAACCGTAAAGGAGC
GAPDH1-R
GTTGCCAAAACCTTCTTAGCCATCC

## Slide 12
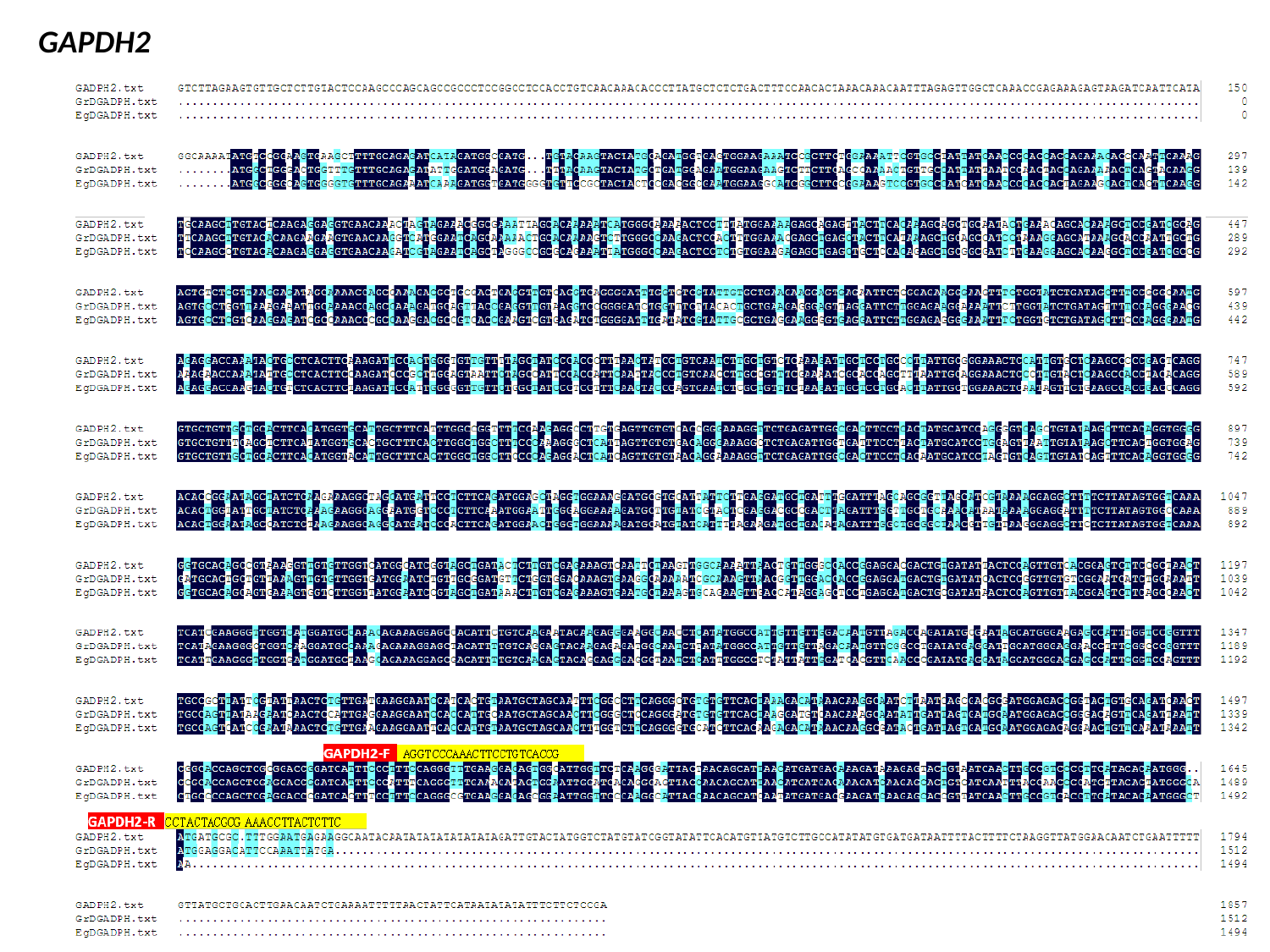

GAPDH2

## Slide 13
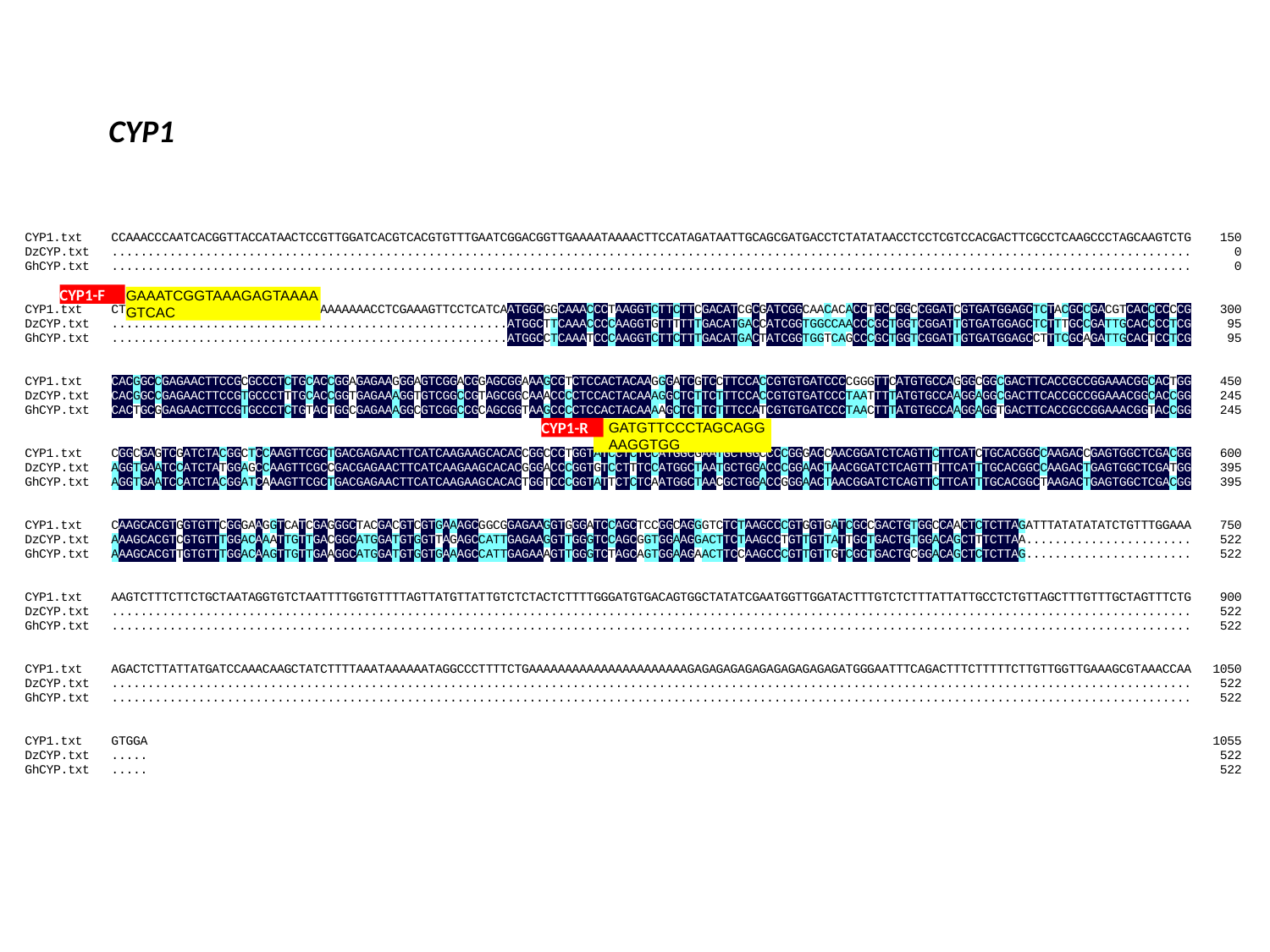

CYP1
CYP1-F
GAAATCGGTAAAGAGTAAAAGTCAC
CYP1-R
GATGTTCCCTAGCAGGAAGGTGG

## Slide 14
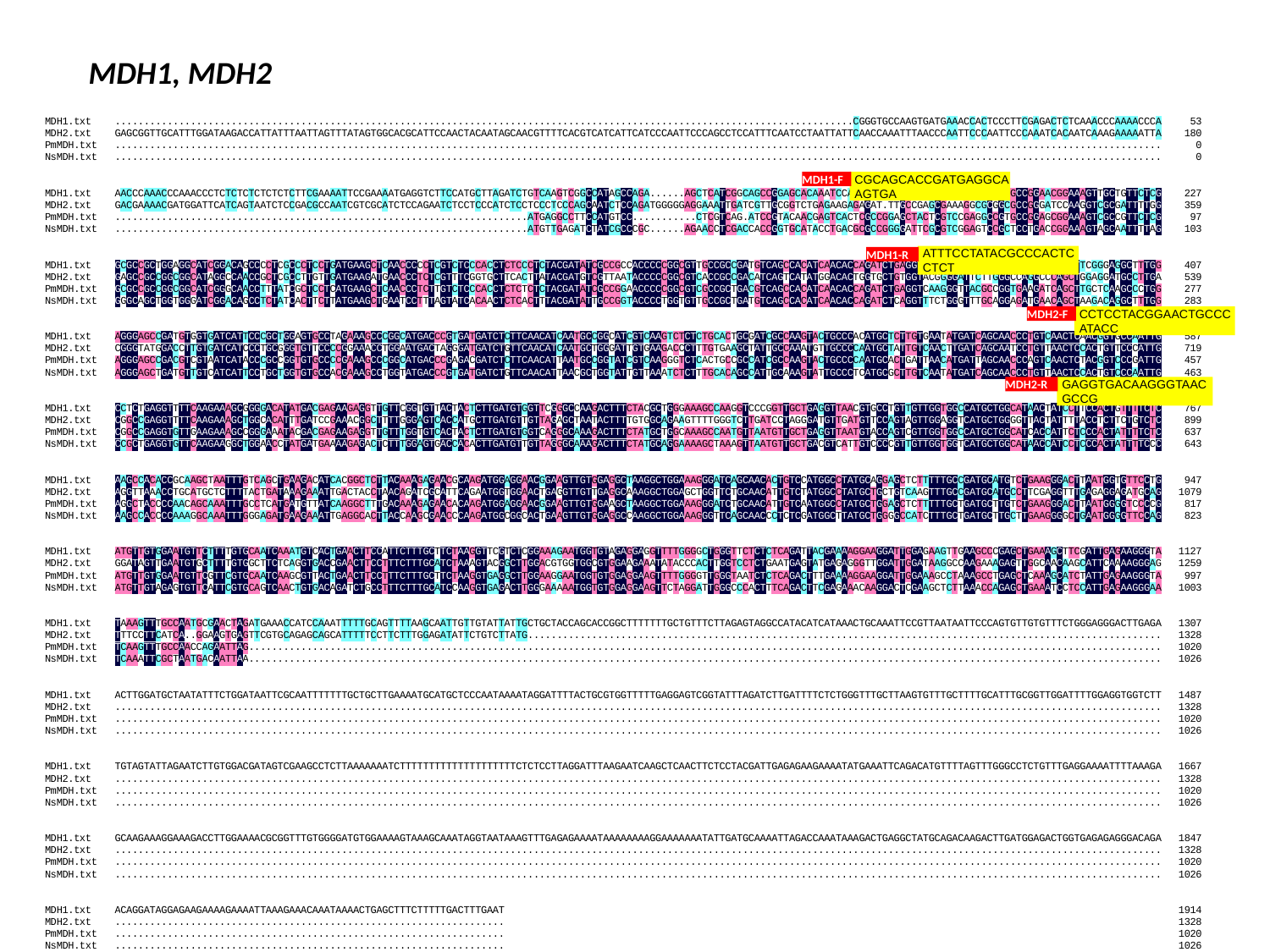

MDH1, MDH2
MDH1-F
CGCAGCACCGATGAGGCAAGTGA
ATTTCCTATACGCCCACTCCTCT
MDH1-R
MDH2-F
CCTCCTACGGAACTGCCCATACC
MDH2-R
GAGGTGACAAGGGTAACGCCG

## Slide 15
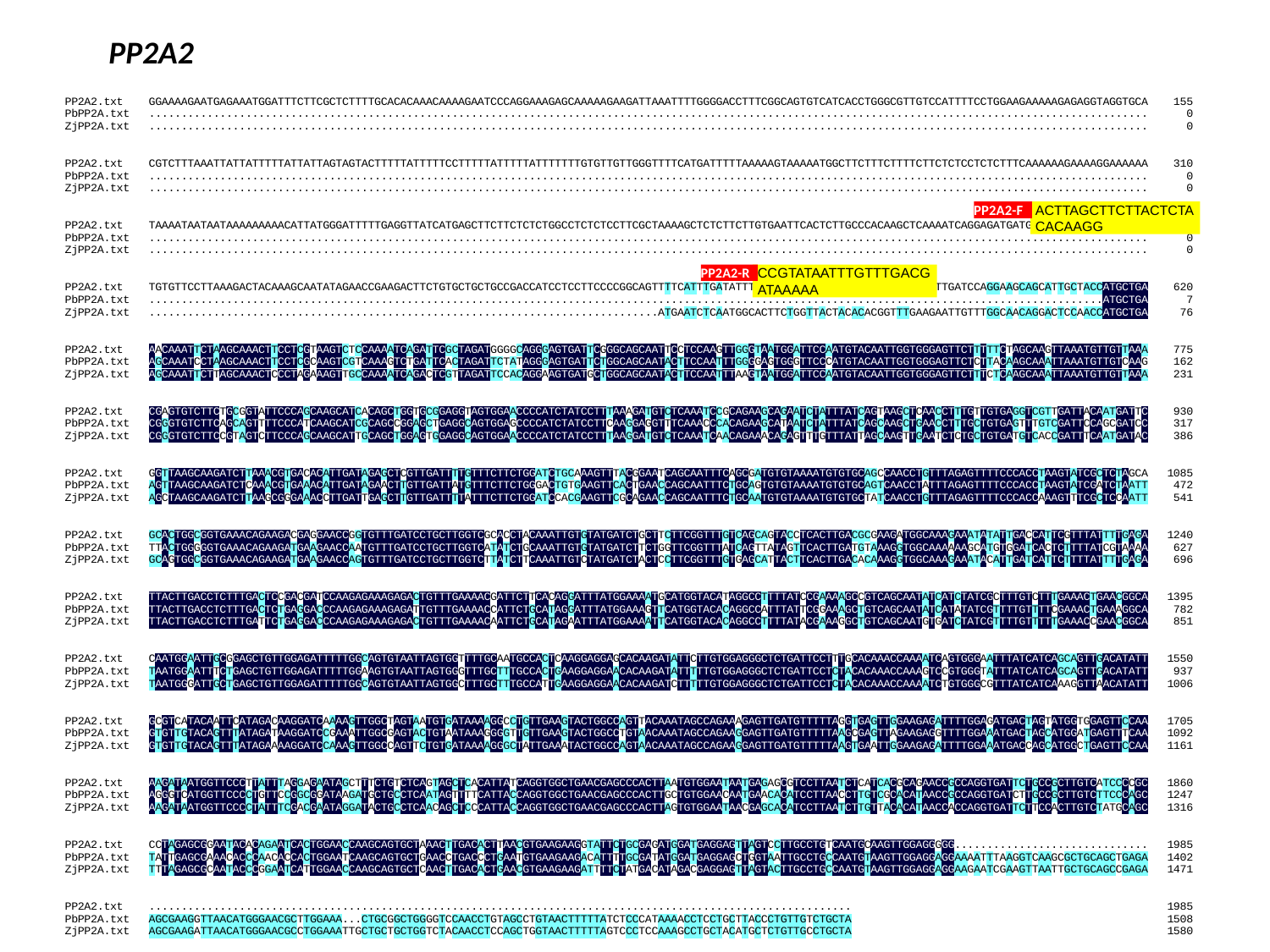

PP2A2
PP2A2-F
ACTTAGCTTCTTACTCTACACAAGG
PP2A2-R
CCGTATAATTTGTTTGACGATAAAAA

## Slide 16
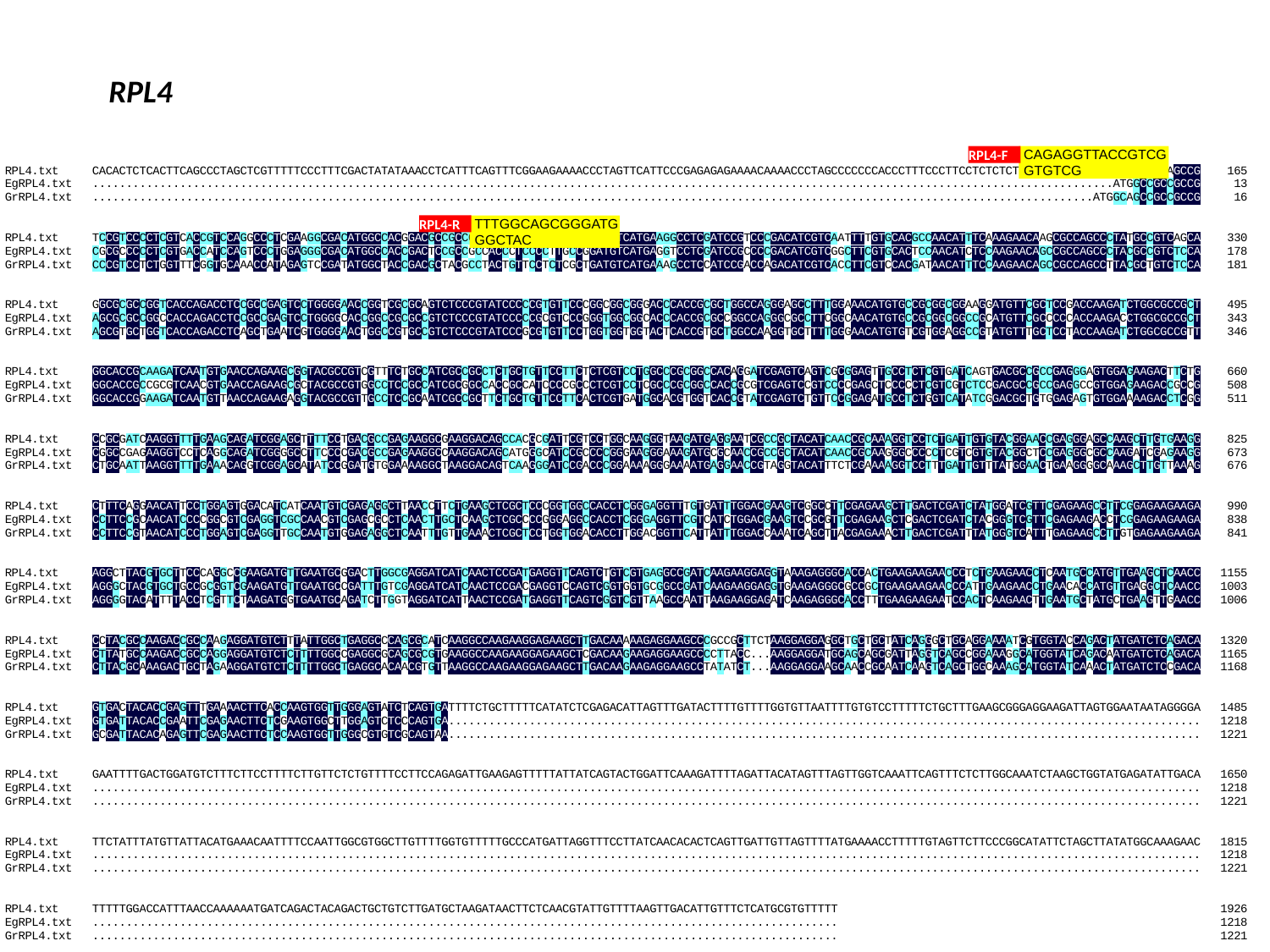

RPL4
RPL4-F
CAGAGGTTACCGTCGGTGTCG
RPL4-R
TTTGGCAGCGGGATGGGCTAC

## Slide 17
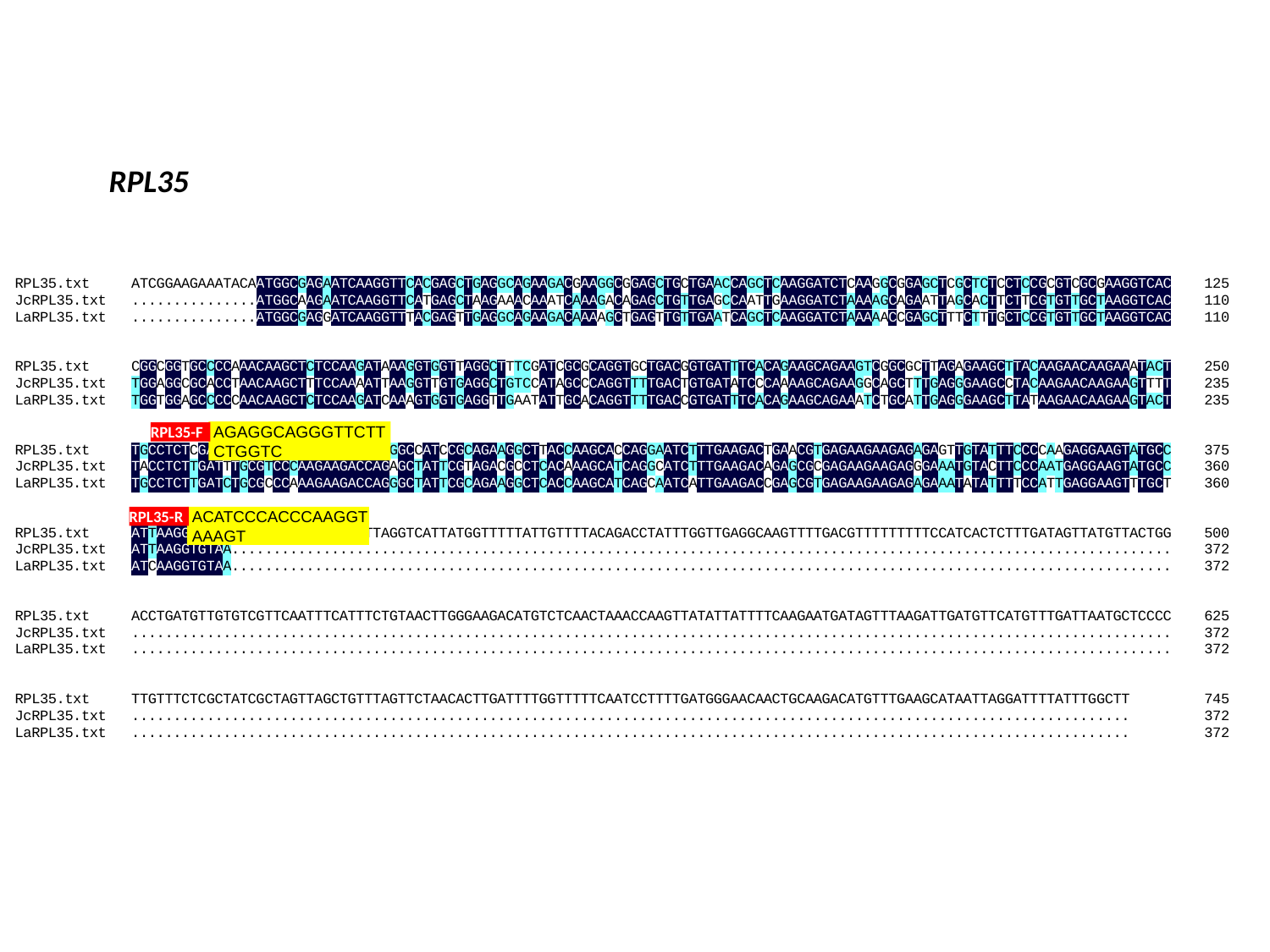

RPL35
AGAGGCAGGGTTCTTCTGGTC
RPL35-F
RPL35-R
ACATCCCACCCAAGGTAAAGT
